# Testing a digitally administered intervention to increase social participation, physical fitness, and health awareness among healthy older adults by means of tablet-based app use: study protocol of the SMART-AGE randomized controlled trial

**DOI:** 10.1186/s13063-026-09641-3

**Published:** 2026-03-21

**Authors:** Nicole Memmer, Meike Snijder-Steinhilber, Heinrich Burkhardt, Claudia Hellmund, Carl-Philipp Jansen, Verena M. Kölsch, Julia Krönung, Beatrice G. Kuhlmann, Lorenzo Masia, Frank Oswald, Barbara Paech, Leon Radeck, Anna Schlomann, Laura I. Schmidt, Marios E. Stefanakis, Anna Wanka, Franziska Kramer-Gmeiner, Melissa Böttinger, Janina Ewert, Sophie Kniepkamp, Katharina Gordt-Osterwind, Elena Litz, Alica Mertens, Uwe Sperling, Hans-Jörg Ehni, Clemens Becker, Hans-Werner Wahl, Anna-Lena Schubert, Tobias Eckert, Jürgen Bauer

**Affiliations:** 1 Network Aging Research, Heidelberg University, Heidelberg, Germany; 2 Department of Psychology, Johannes Gutenberg University Mainz, Mainz, Germany; 3 Medical Faculty Mannheim, Heidelberg University, Heidelberg, Germany; 4 Geriatric Centre, Mannheim University Hospital, IV. Medical Clinic, Mannheim, Germany; 5 Geriatric Center, Medical Faculty Heidelberg, Heidelberg University, Heidelberg, Germany; 6 Department of Clinical Gerontology and Geriatric Rehabilitation, Robert Bosch Hospital, Stuttgart, Germany; 7 University of Hagen, Hagen, Germany; 8 School of Social Sciences, Department of Psychology, University of Mannheim, Mannheim, Germany; 9 Department of Computer Engineering School of Computation, Information and Technology, Technical University of Munich (TUM), Munich University, Munich, Germany; 10 Carl von Ossietzky University, Oldenburg University, Oldenburg, Germany; 11 Institute for Computer Science, Heidelberg University, Heidelberg, Germany; 12 Institute for Educational Sciences, Heidelberg University of Education, Heidelberg, Germany; 13 Institute of Psychology, Heidelberg University, Heidelberg, Germany; 14 Clinic for Trauma and Reconstructive Surgery, University Hospital Heidelberg, Heidelberg, Germany; 15 Institute of Sports and Sports Sciences, Heidelberg University, Heidelberg, Germany; 16 Institute for the Ethics and History of Medicine, University of Tuebingen, Tuebingen, Germany

**Keywords:** Digital based intervention, Social participation, Physical fitness, Health awareness, Technology acceptance, User-centered design

## Abstract

**Background:**

Digital interventions for older adults may significantly extend preventive action to postpone disability and preserve health-related quality of life. However, more evidence is needed from multi-domain interventions using broad-scale objective and self-report assessments and intra-individual change data-analytical techniques.

**Method:**

SMART-AGE examines the effect of an app-based multilevel treatment designed to enhance social participation, physical fitness, and health awareness. The target population comprises healthy and community-dwelling adults 67 years and older with basic digital skills in two socially diverse communities. Treatment relies on an Android-based tablet computer, on which three apps offering interventions in the core areas of social participation, physical fitness, and health awareness are pre-installed. A feedback app designed to provide participants with a feedback option at any time is also offered. Participants are randomly assigned to three intervention arms and assessed at baseline and after 3 and 6 months. Arm 1 receives the full intervention, consisting of the social participation app, the physical fitness app, the health awareness app, and the feedback app. The health awareness app is available in months 4 to 6, meaning that participants receive the full three-app intervention only in the second half of the intervention period. Arm 2 receives the social participation app and the feedback app throughout the intervention. Arm 3 serves as an active control condition in that a stand-alone tablet with a low-dose introduction to publicly available standard apps is provided. The data protocol includes assessment of three primary outcome domains: social support and loneliness, motor capacity and physical performance, and health awareness and health locus of control. Potential moderators (e.g., cognitive function, depression) as well as various technology-oriented constructs (e.g., skills, acceptance) are also assessed. App use data are automatically collected across the full intervention interval in arms 1 and 2. Data management is conducted within a cloud-based REDCap architecture. Feedback recordings via the feedback app are collected in arms 1 and 2 and undergo qualitative analysis.

**Discussion:**

The SMART-AGE intervention aims to enhance core domains of health-related quality of life in community-dwelling older adults through an app-based multi-domain intervention and a user-centered approach.

**Trial registration:**

German-Clinical-Trials-Register, DRKS00034316. Registered 29-May-2024, https://drks.de/search/en/trial/DRKS00034316. The study’s design and hypotheses were also pre-registered in the Open Science Framework (OSF) prior to study enrollment (10.17605/OSF.IO/YQEBW, 2023–04-28).

**Supplementary Information:**

The online version contains supplementary material available at 10.1186/s13063-026-09641-3.

## Introduction

The randomized controlled trial “Smart Aging in Community Contexts: Testing Intelligent Assistive Systems for Self-regulation and Co-regulation under Real-Life Conditions” (SMART-AGE) aims to strengthen the evidence on app-based multi-domain interventions as a large-scale preventive tool for healthy older adults. This accords well with the broader digital transformations among older populations, which promise significant benefits but also involve potential challenges and risks [1–3].

## Background and rationale

SMART-AGE is designed as a complex intervention trial (see also [4]) that targets three intervention domains (social participation, physical fitness, health awareness) in various combinations. An increase in social participation is defined as enhanced social support, reduced feelings of loneliness, and an expansion of social networks. Improvements in physical fitness are reflected in increased muscle strength, better balance, and enhanced gait control. An increase in health awareness is understood as improved self-monitoring of health and stronger beliefs in one’s own ability to influence health through personal behavior (i.e., internal health locus of control). In addition, a direct effect of app use on technology skills and technology attitudes is expected.

**Fig. 1:**
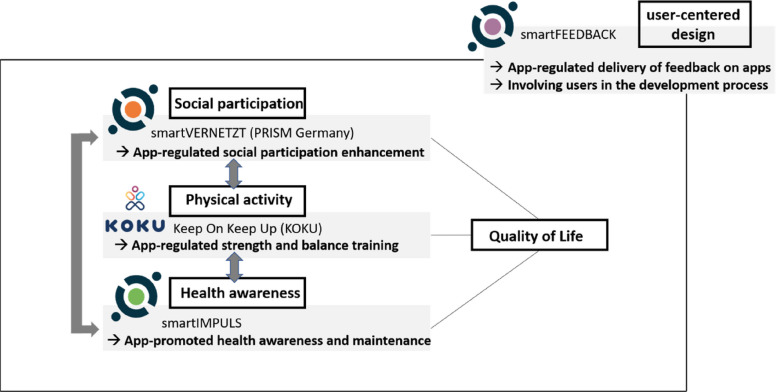
Overview of the app-based multi-domain intervention package in SMART-AGE

SMART-AGE’s conceptual background for doing preventive app-based multi-domain interventions builds on the World Health Organization’s (WHO’s) well-established model of the International Classification of Functioning, Disability and Health (ICF) [5]. According to the ICF, preventive interventions should primarily support older adults in maintaining or regaining their ability to function. Functioning here means being able to use one’s physical and mental capacities in daily activities and social life, always in interaction with the surrounding environment. Digital technologies offer an increasingly important component of facilitating environments in the lives of older adults able to enhance and optimize functioning [6–8]. Building on the ICF as a framework for public health prevention, we assume that enhancing social participation, physical fitness, and health awareness via digital interventions constitutes an important strategy to support quality of life (QoL) in older age [9]. We combine in SMART-AGE the strengthening of these critical resources with a user-centered design approach in that we engage participants in systematic app-based feedback loops along the full intervention period (Fig. 1).

### Digital strategies for enhancing social participation

Sustaining social participation and relations while mitigating loneliness in older adults is of paramount importance for QoL in later life [8, 10]. Social factors are integral to healthy life expectancy at large, as they are associated with a decreased risk of depression [11], dementia-related disorders [12], premature mortality [13], and enhanced well-being and satisfaction with life [14]. Furthermore, established theories of behavior change emphasize the pivotal role of social factors, including support and connectedness, in both initiating and sustaining behavioral changes as well as in promoting cognitive performance [15–17]. In the field of digital apps promoting social participation the “Personal Reminder Information and Social Management System” (PRISM), developed by Czaja et al. [6] stands out due to its breadth of social domains targeted as well as the rigor of evaluation. In a randomized controlled trial involving older adults aged 65 years and above, who lived alone with minimal prior computer and internet use, PRISM users demonstrated a significantly greater reduction in loneliness (*d* = 0.17) and a more pronounced increase in perceived social support (*d* = 0.28) compared to the control group after 6 months. Moreover, PRISM users exhibited a notable enhancement in computer proficiency (*d* = 1.11). SMART-AGE also builds in the area of social participation on additional previous research as work by Kawaguchi et al. [18] and Neal et al. [19]. In SMART-AGE, a PRISM version adjusted to German conditions, named smartVERNETZT, is deployed.

### Digital strategies for enhancing physical activity

Besides its most important role for social participation, enhanced physical fitness in later life comes with multi-level positive effects on various health domains (e.g., cardio-vascular, cognitive), maintenance of functional independence, as well as supporting fall prevention [20, 21]. Meanwhile, a range of reviews and meta-analyses showed that app-based mobile interventions are effective and highly promising for altering physical fitness and activity [22–24]. Additionally, digital apps contribute to improving accessibility, scalability, cost-effectiveness, and equity in healthcare [25]. A prime example is the app Keep On Keep Up (KOKU). Previous studies on KOKU demonstrated that users found the program appealing, motivating, and entertaining after 6 weeks of independent and unsupervised usage [26]. SMART-AGE also considers the evaluative studies conducted by Daniels et al. [27] and van Het Reve et al. [28]. In the SMART-AGE study, a German version of KOKU is used.

### Digital strategies for enhancing health awareness

Apparently, issues of health literacy, health awareness, and health self-monitoring are critical for health maintenance, prevention, and health-promoting behaviors. In agreement with WHO’s view on healthy aging [29], the process of enabling older individuals to take control over their health is key for SMART-AGE’s approach to health. It could be demonstrated that digital tools, especially digital self-monitoring systems, have the potential to support health literacy and awareness [7]. Initial evidence has shown that the use of these tools by older adults is feasible and goes along with a positive effect on health behavior and the management of (chronic) diseases and multimorbidity [30–32]. Primary outcomes for app-based interventions in this area include health locus of control, health knowledge, and motivation to monitor one’s health. The smartIMPULS app is implemented for these purposes in the SMART-AGE study.

### Digital strategies for enhancing feedback provision in a user-centered design with participatory design approach

By systematically focusing on the needs, priorities and critique of the target group, the likelihood that apps can be developed that promote high user acceptance and compliance is much increased [33–35]. In contrast to traditional methods such as focus groups and structured interviews, which are also integral to the user-centered design process, real-time feedback may better capture the challenges users face during actual usage [36, 37]. Doing so, a newly designed app (smartFEEDBACK) aims to bolster the user-centered design approach by real-time feedback generation across the full trial length. Details and functionalities of the apps used in SMART-AGE are provided below.

In conclusion, previous research has yielded encouraging findings on digital interventions, providing a solid foundation for SMART-AGE. However, most previous research took a single-domain approach, thereby overlooking the potential effects of combining different digital apps. For instance, while app-based interventions have been successfully evaluated previously in domains such as physical activity [e.g., [27, 28]] or social participation [e.g., [6, 18, 19]], little is known about the effects of their integration within a multi-domain framework.

## Objectives

According to our preregistration (see below), objectives of the SMART-AGE trial are as follows: Can the regular use of the offered apps (1) prevent or reduce loneliness and promote social support (particularly smartVERNETZT), (2) improve motor capacity (particularly KOKU), (3) increase health self-awareness and health-related control beliefs (particularly smartIMPULS), and (4) increase technology-related skills and the acceptance of technology?

To address these objectives, we decided for an intervention scheme as follows: a full intervention group (IG), a partial IG, and an active control group (CG). An active CG was installed to ensure that observed effects can be attributed to app use and not simply to the provision of a tablet to the participants coming with some social attention. In the full intervention condition, the participants will have access to all of the SMART-AGE specific apps, namely smartVERNETZT, KOKU, smartIMPULS, and smartFEEDBACK. In the partial intervention condition, the participants will have access to two SMART-AGE specific apps, namely smartVERNETZT (containing all standard apps) and smartFEEDBACK. This arm allows us to investigate the impact of the smartVERNETZT app without the co-use of other apps that also aim to improve the quality of life. It also serves as a most direct comparison to the previous PRISM trial [6]. Participants in the active CG will just be given the device and receive a basic introduction to publicly available and pre-installed standard apps (e.g., calendar app, e-mail app). In all of the three treatment arms, participants will receive a tablet and be given a short introduction on how to use a tablet in general.

Hypotheses based on these treatment variations are as follows:

1. Participants who use a tablet equipped with the smartVERNETZT app (full and partial IG), which encourages social networking and participation, are expected to experience lower levels of loneliness and higher levels of social engagement compared to those using a tablet with only standard applications. This effect is expected to be even larger in the full IG than in the partial IG due to the synergistic benefits derived from using all apps designed to enhance quality of life.
2. Participants who are in the full IG (who all have access to smartIMPULS) have increased health-related self-awareness and health-related control beliefs, compared to the active control and the partial IG, who both do not have access to smartIMPULS. Between the latter two groups, we do not expect any differences in health-related self-awareness and health-related control beliefs.
3. Participants who are in the full IG (who all have access to KOKU) will show better gait performance and motor functioning, compared to the active control and the partial IG, who both do not have access to KOKU. Between the latter two groups, we do not expect any differences in gait performance and motor functioning.
4. Over time, participants using a tablet will report greater technology acceptance and develop technology-related skills than before using a tablet. This increase in technology acceptance and technology-related skills is larger for participants in the full and partial IG. In addition, exploratory analysis will give more insight into the dose–response relationship between the amount of apps and their impact on technology acceptance and technology-related skills***.***

## Method: participants, interventions, and outcomes

### Trial design

**Fig. 2:**
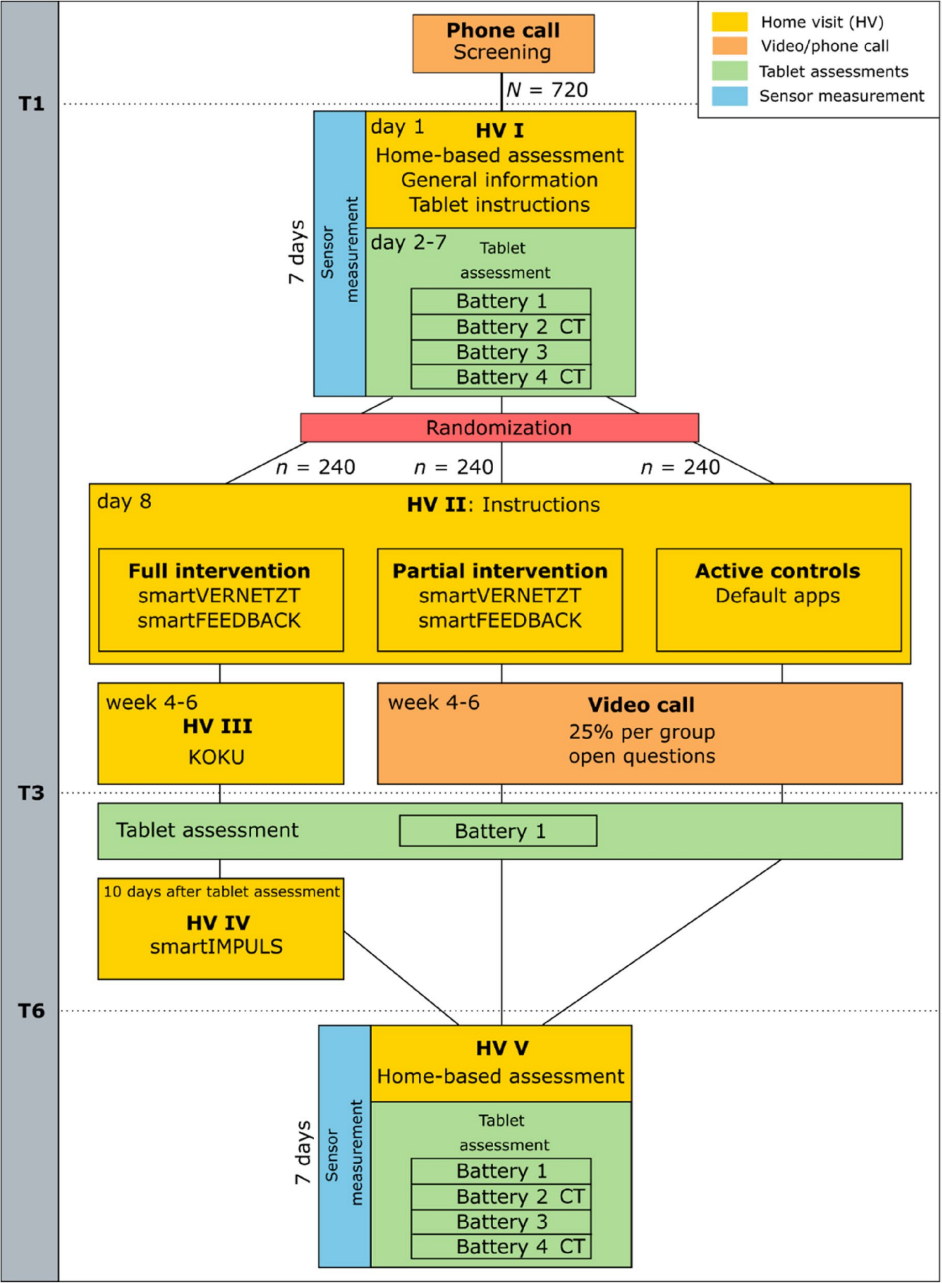
Overview of SMART-AGE design. Note:* N* = total sample size, *n* = sample size in the treatment arms, CT = cognitive tasks, T1/T3/T6 = time point in study

The study is a three-armed, randomized controlled trial with repeated measures over 6 months. Treatment relies on an android-based tablet computer, on which three apps offering structured interventions in the three core areas of social participation, physical fitness, and health awareness are pre-installed. Also, an app designed to provide study participants with a structured feedback option at any time of the trial is offered. Participants are randomly assigned to three intervention arms and assessed at baseline and after 3 and 6 months. Arm 1 receives the full intervention, consisting of the social participation app, the physical fitness app, the health awareness app, and the feedback app. The health awareness app is available in months 4 to 6, meaning that participants receive the full three-app intervention only in the second half of the intervention period. Arm 2 receives the social participation app and the feedback app throughout the intervention. Arm 3 serves as an active control condition in that solely a stand-alone tablet implementation with a low-dose introduction to publicly available standard apps are provided. The trial protocol follows the Standard Protocol Items: Recommendations for Interventional Trials (SPIRIT) [38]. A completed SPIRIT checklist is provided in the Additional file. A detailed overview of the study procedure is provided in Fig. 2.

### Participant timeline

Home-based assessments take place at the time of study enrollment (T1) and after 6 months (T6). In addition, a brief online self-assessment will be conducted 3 months after study enrollment (T3). At the beginning of the first home visit (HV; day 1 in T1), participants are informed about data protection, can ask open questions, and get general information about the study. Then they are provided with a tablet, a corresponding manual, and are instructed on how to use the tablet in a short training session. At that moment, the tablet is switched to “study mode,” enabling the monitoring of app usage data of the participants from that point forward. Afterwards the online-assessments are explained and set up on the tablet. Then the home-based assessments take place including cognitive and motoric measurements. During the motor capacity assessment, the body-fixed movement sensor is placed on the participant’s lower back.

One week after the initial HV, the randomization has already taken place, and a second HV is conducted for participants of all groups. During this visit, the tablets are modified allowing participants in both IGs (arms 1 and 2) access to two SMART-AGE specific apps: smartVERNETZT and smartFEEDBACK. These groups also receive instructions and a manual on how to use these new apps. Participants in the CG (arm 3) are given access to the standard applications on the tablet. However, detailed explanations are only given for the video chat tool. The documents containing information about the SPs, completed and signed during the HVs, as well as the data sheets of the cognitive test battery, are securely stored in a locked cabinet in SMART-AGE designated office spaces. Four to 6 weeks after the second HV, participants in the full IG additionally receive access to and are instructed in the use of the KOKU app. Approximately 10 days after the digital assessment at 3 months, the participants of the full IG are introduced to the smartIMPULS app during HV IV.

In order to counterbalance potential social activation effects, 25% of participants in study arms 2 and 3 (active CG and the partial IG) were randomly selected to receive one additional video calls. Randomization was performed using the R package “blockrand” [39].

Finally, it deserves mentioning that intervention schemes are implemented without altering usual care pathways, which will continue without any change in both trial arms. There are no restrictions regarding concomitant care provision or change during the trial.

**Fig. 3:**
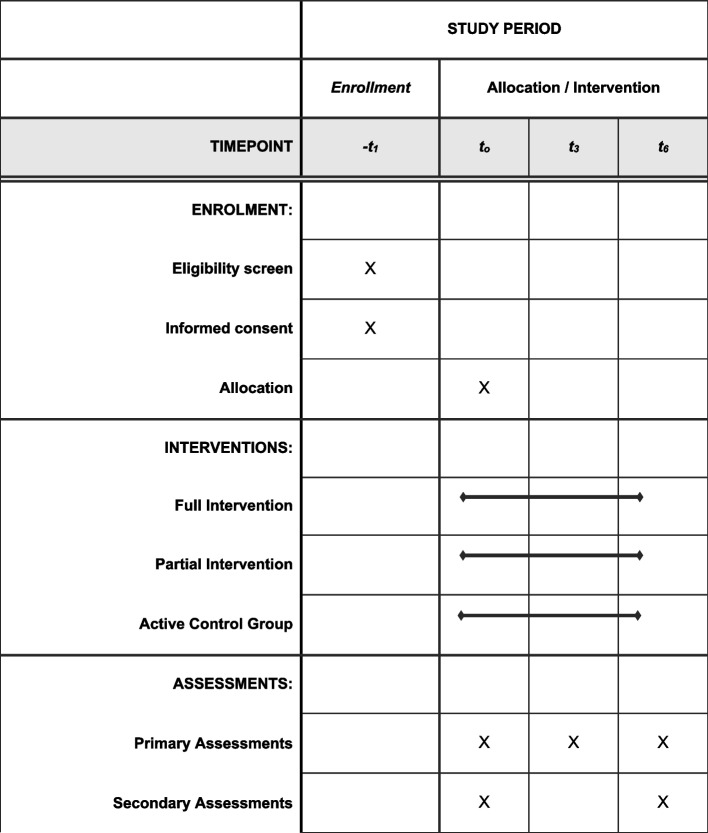
Standard Protocol Items: Recommendations for Interventional Trials (SPIRIT) figure (numbers indicated under the heading “time points” represent month)

For a full overview of SMART-AGE trial components, see checklist of SPIRIT provided in Fig. 3.

### Trial setting

The SMART-AGE trial addresses community-dwelling older adults in the cities of Heidelberg and Mannheim (Germany). Cities were selected due to their nearness to the university’s study site and socio-structural differences. Heidelberg is a since long-time established university city with overall relatively high socio-economic level, limited within-community heterogeneity, and low migration rate. In some contrast, Mannheim comes with overall relatively lower socio-economic level, pronounced within-community heterogeneity, and high migration rate.

### Eligibility criteria

Interested individuals who contact the study team undergo a screening process over a phone call to determine if they meet specific criteria for inclusion or exclusion from the study. Individuals who are younger than 67 or living in a nursing home, have severe cognitive limitations, no internet access, no PC/Tablet experience, severe medical conditions, severe visual/hearing impairments, no/poor knowledge of German, or work 20 + hours per week are excluded.

### Intervention

SMART-AGE’s digital intervention builds to a large extent on previously successfully evaluated tools [6, 40]. In this context, smartVERNETZT represents the German adaptation and further development of the successfully tested PRISM system [6]. The app KOKU also underwent intensive testing within British public health improvement efforts [26, 40]. The app smartIMPULS represents the translation of an earlier system completely focusing on geriatric patients to healthy older adults (project: SINQ-Service integration and network management to improve social coexistence of geriatric patients in the community [41]). All apps underwent in SMART-AGE additional testing to assess their usability and feasibility. Focus group interviews with nine participants who used smartVERNETZT and smartFEEDBACK for at least 7 days indicated that the app was perceived as engaging and user-friendly [42]. Internal pilot testing of KOKU confirmed previous findings [26], suggesting that the app was perceived as easy to use, useful and enjoyable. Given the new development of smartIMPULS based on an earlier geriatric patient version, a 6-week pilot study with 30 participants was conducted, in which the system achieved a score of approximately 77 in the System Usability Scale [43], which indicates good usability [44]. Based on feedback in pre-studies, the user surface and content of the apps were further optimized.

The programming of the apps smartVERNETZT, smartIMPULS, and smartFEEDBACK was conducted by the Institute of Computer Science at the University of Heidelberg. The apps are accessed via a tablet (Lenovo M10 FHD Plus). Studies indicate that tablets are well-accepted by older individuals due to their intuitive gesture control and convenient size [45].

The usage of the apps is monitored, and in case of 2 weeks of non-use, a single reminder email is sent to the tablet. In addition to digital and analog user manuals and instructional videos, a helpline (via email and phone) has been established. For details on data privacy and security measures related to the apps, please refer to the section “Data storage, data processing, and data quality.” Images of the apps are provided in the supplement (see Appendix-A1). Table 1 provides an overview of the contents of apps used.

**Table 1:** Overview of Contents of Apps Used in SMART-AGE

Name of app	App feature/content
smartVERNETZT	– Weekly news on findings on physical activity, technology, social participation and local events – Integrated tools: calendar, address book, camera, notes, clock, calculator, Internet browser and games are included – Provides links to health websites, local counseling services, digital literacy resources, cognitive attention games, analog exercise programs, activities and offers (e.g., group meetings, volunteering), and local educational programs – Allows social networking through e-mail, social networks, and video chat
KOKU	– Progressively structuredtraining program comprising seven levels, each lasting a minimum of 2 weeks. Each week includes 3 training days, with three exercises per day; Green check marks indicate progress – Gamified training with an interactive avatar, demonstrating 26 strength and balance exercises (with goals description and safety instructions), based on evidence-based fall-prevention programs (Otago, FaME [46, 47]) – Users provide feedback on physical exertion; based on this training progression is automatically adjusted – Four health literacy games on home safety, bone health, nutrition and hydration
smartIMPULS	– Daily prompts with one to four questions addressing current life, functioning in daily life tasks and health situations – Addresses the following health domains: nutrition, activities of daily living, mobility, social participation, cognitive functioning, physical functioning, psychological factors, preventive health check-ups, pain, fear of falling, perceived stress, hearing ability, and vision – Questions repeat at set intervals based on expected changes in each domain; unanswered questions accumulate but can be skipped – Supports attention to specific health domains by giving hints based on user responses – Hints suggest follow-up actions (e.g., doctor visit, counseling) or offer additional information – Provides an overview of given answers, filterable by health domains and received hints, including status indicators (new, postponed, completed)
smartFEEDBACK	– Allows users to provide feedback on app use and usability issues at any time – Supports both explicit and implicit feedback, either voluntarily submitted (push) or prompted by the system (pull) – Up to five feedback questions per day (pull); all questions can be skipped

### Patient and public involvement

Several steps were undertaken to secure active involvement of participants. First, we referred in all personal interactions with participants as well as all written study documents to participants as study partners to promote an open and mutual appreciation communication style. Second, the apps were developed and refined based on findings from prior pilot studies conducted with similar target groups. Third, a feedback app specifically designed by SMART-AGE software engineers was used during the full trial to collect detailed feedback from study partners using the apps. Finally, post-study events are planned to share results and gather input for future research.

In terms of dissemination, SMART-AGE not only aims for scientific publishing at the highest quality levels possible but also strives to translate its findings to the community ecology which serve as study platforms and hopefully later beyond. To make this come true, SMART-AGE invests in continuous contact and information exchange with community authorities, local senior organizations, and the general medical community. More specifically, SMART-AGE will do two intensive feedback meetings separated by community. Also, a public event for presenting the main findings is envisaged in cooperation with city authorities involved. We also have definite plans for a senior citizen workshop to further discuss the ethically oriented findings of SMART-AGE. SMART-AGE also is in contact with Heidelberg University’s Innovation Hub to prepare a possible dissemination of further improved apps in a later set.

### Outcomes

There are four primary outcome domains in this study that will be compared across all three study arms:

1. Health-related self-awareness, control beliefs, and self-efficacy, which includes measures like the Health Locus of Control Scale [48], Health Literacy [49], Adapted Health Consciousness Scale [50], and Self-efficacy [51];
2. Motor capacity and performance, which includes measures like the Timed Up and Go Test [52] and movement sensor-based physical activity parameters (e.g., steps per day) using Axivity AX6 sensors;
3. Loneliness and social support, which includes measures like the UCLA Loneliness Scale [53], Lubben Social Network Scale [54].
4. Technology acceptance, competence, and usage, which includes measures like Computer Anxiety [55] and End User Frustration [56].

Another important part of the assessment architecture in SMART-AGE is the generation of multiple usage data while the apps are used by study partners. This includes, for instance, the assessment of weekly usage frequency and intensity (e.g., total time spent using the apps, number of interactions within the apps), as well as the analysis of app-specific features used—such as whether video calling was utilized in smartVERNETZT, how many training levels were completed in KOKU, or how many questions were answered in smartIMPULS or smartFEEDBACK. These metrics will be analyzed over time and across multiple apps to identify usage patterns and dynamics, which can then be related to changes in the targeted outcomes.

A complete list of all assessments, encompassing all measures related to the main and secondary hypotheses, as well as demographic measures that describe the sample is provided in Appendix Part 2. In addition, we also collect objective user data from the SMART-AGE apps to evaluate in more detail the app usage.

### Sample size

The sample size was determined through an a-priori power analysis to ensure adequate power for the planned structural equation modeling analyses. We will study the effect of the intervention groups as contrasted with the active control condition on main outcomes using latent change models, in which each of the four main outcome domains will be represented in latent space by at least three manifest variables. Assuming measurement invariance across the three study time points, the two intervention sites, and the two experimental groups and one active control group, this results in a model with 128 estimated parameters and 16,162 degrees of freedom. With the requirement of five participants per estimated parameter, an expected dropout rate of 10% and a further increase to balance the strata, we plan to collect a sample of 720 participants to be analyzed based on an intention-to-treat analysis, that is, all participants having entered the study at baseline will become part of the statistical effect analysis independent of their “fate” along the full observational period. Moreover, at each of the three study time points for each of the three groups, the planned sample size meets Browne and Cudeck’s [57] recommendations for model testing based on the close fit hypothesis (H0, ε ≤ 0.05; H1, ε ≥ 0.08; *α* = 0.05) using the root mean square error of approximation (RMSEA; 44). This provides a statistical power of 1 – *β* > 0.99 for the main model across all study time points as well as for analyses conducted at a single time point, ensuring that the study has sufficient power to reject the model if its fit to the empirical data is inadequate.

### Recruitment

The study coordinators obtain addresses from the city registries of Mannheim and Heidelberg to invite potential study partners with a letter of information and detailed study information. After the positive screening and the first HV, the participants are assigned to one of three study arms through block randomization and are stratified with respect to their place of residence (Heidelberg or Mannheim), age group (67–74 years or ≥ 75 years), 1and gender (male or female). The randomization sequence was generated by an independent statistician and implemented via a secure, web-based system. Each group consists of *N* = 240 subjects (720 in total). We plan to recruit 720 individuals who are at least 67 years old and will be equally assigned to the three study arms. We will study the effect of the experimental group on primary outcomes using latent change models, in which each of the four primary outcomes will load onto at least three manifest variables.

We use chronological age as a proxy for different digital skill levels, chronic conditions prevalence, and diverse cohort socialization. To counterbalance an expected and undesired oversampling of those in the 67 to 74 years of age range, we started at the beginning of the last third of the enrollment period to only sample those in the old-old age range. Gender is used as a stratification because differences between genders have been found in that males revealed higher digital skill levels and females used socially oriented internet activities more often [58].

The recruitment process is assessed every 3 months to monitor the target rate of approximately 15 participants per week–if recruitment falls behind, we consult the advisory board for alternative strategies for recruitment and advertising.

To ensure a certain level of diversity, a two-fold recruitment strategy was applied. First, participant recruitment was based on random sampling of addresses provided by local city registries. Due to German data protection regulations, researchers were only allowed to send out initial invitation letters without follow-up reminders. To lower the participation threshold, the invitation package included a letter of support from the city mayors, framed the study as a unique learning opportunity, and offered the intervention tablet as an incentive. Second, sampling was conducted in two socioeconomically contrasting cities: one, a historically preserved university city; the other, a traditionally working-class city with significant World War II destruction and one of the highest proportions of migrant populations in Germany.

We will include as many participants as possible until the end of August 2024. If we do not manage to obtain our sampling goal of 720 participants, we will, following what has been said in the study’s preregistration, consult the advisory board and recruit as many participants as needed to reach the next multiple of 72 to balance the eight strata.

### Blinding

Participants are always aware of the study arm they are assigned to, as they either have access to none, two, or all four of the project apps, except during the baseline assessment. To gather data, two HVs, HV I and HV V, are conducted. It is crucial that the researchers conducting these visits remain blinded to the participants’ group assignments. To facilitate this, the research team is divided into two distinct groups: assessors and app-instructors. The assessors are responsible for data collection during HV I and HV V, while the app-instructors handle HV I, II, III, and IV. The initial HV occurs prior to the randomization of participants, allowing both assessors and instructors to be involved in this stage of the study. We do not anticipate any requirement for unblinding the assessors, but if required, the project coordinators will have access to group allocations and any unblinding will be reported. The randomization list used to allocate participants to the study arms was created by the lead study statistician and the project coordination team, who are the only ones with access to the data file containing personalized information. After individuals are screened positive, the project coordination allocates them to the randomly selected study arm using a study management software. The software allows the information of the allocated study arms to be hidden from blinded assessors while showing it to user groups who need this information.

## Methods: data collection, management, and analysis

### Data collection methods

At each of these two time points (T1 and T6), trained researchers will visit participants at home and conduct a number of assessments (see Appendix–A2), including a battery of cognitive tasks and the application of state-of-the-art body-fixed movement sensors (Axivity AX6, Axivity Ltd, Newcastle upon Tyne) to measure physical activity for 7 consecutive days. Moreover, participants will be asked to complete a set of web-based questionnaires using their tablet within a week of each of these visits by means of a RedCap data architecture. Web-based questionnaires are sorted into four sets of comparable length that each take no longer than an hour to complete. Participants will be asked to respond to one set of questionnaires per day. After the second and fourth set of questions, participants perform further cognitive tasks on the tablet. The first set of questions, presented on the first day, includes the primary outcome measures and is repeated at T3 in order to have a higher granularity of primary outcome data.

### Data management

Data storage and data processing are subject to the highest standards of data security. First, no personally identifiable data is processed outside the EU. Second, all questionnaire-based online assessments are conducted using a REDCap instance hosted on a secure cloud at Heidelberg University, ensuring strict compliance with data security requirements. The SMART-AGE apps are also located in the same secure cloud system. Third, the REDCap data and the app data are exclusively transmitted in encrypted form from the tablets to the respective cloud server. Subsequently, the data is securely transferred to a high-security research data server, also located within the University of Heidelberg. This server is the central location to which all data from the main project (including the data from the sensors, the apps, and the cognitive assessments) and, if possible, the data from the sub-studies are transferred.

All analyses with pseudonymized data must be performed directly on the server. Each researcher can only access the specific data they are directly responsible for, for the purpose of monitoring data quality or to which they have been granted access by the principal investigators. This personalized access is provided after an internal multi-stage review process of the submitted statistical analysis plan.

Constant supervision and analysis of the multilevel data infrastructure is an essential part of the lead statistician’s work package. To achieve high data quality and immediate data access, online questionnaires orchestrated by the software REDCap are used to a large extent to minimize data entry errors. Closed question formats are mainly used and when an open format for quantification is necessary, plausible value ranges are specified in advance to detect invalid values. A data preprocessing script undergoing continuous updating was generated in advance in the programming language R to correct defined mistakes in the data, such as misspelled IDs or duplicate entries created due to internet connection problems.

The de-identified data set of the SMART-AGE study will be shared in a secure data repository of Heidelberg University after the completion of the study.

### Statistical methods

Our main hypotheses will be tested using multilevel and structural equation models, following an intention-to-treat analysis. We will test the effect of experimental group on primary outcomes separately for each outcome with a 3 × 3 factorial design multilevel model with the between-subjects factor *study arm* (active CG vs. partial IG vs. full IG) and the within-subjects factors *study time point* (study start vs. 3 months vs. 6 months), followed by post-hoc comparisons controlling for the family-wise error rate. Models will include random intercepts for each participant and their place of residence. As covariates, we will consider participants’ gender, their age, and their frequency and intensity of app usage. We plan to report all analyses with and without the inclusion of these covariates. Where the assessment of primary outcomes is only possible during HVs, we will remove the 3-months level from the within-subjects factor *study time point*.

In addition, we will test the effect of experimental groups on primary outcomes first separately for each outcome and then jointly for all four primary outcomes using latent change models. In these models, the multilevel structure of the data will be accounted for by multigroup comparisons to examine whether (a) the effects of the two IGs are larger than the effects of the active CG (see hypotheses), whether (b) the effects of the full IG are larger than the effects of the partial IG (see hypotheses), and (c) by which individual and/or sociodemographic variables the effects of the two interventions on the four primary outcome variables are moderated.

For all statistical tests, we will use alpha = 0.05 as the threshold for statistical significance. Post-hoc comparisons will be controlled for the family-wise error rate. We will be reporting standardized effect sizes and confidence intervals whenever possible. In addition, we will evaluate the goodness-of-fit of SEMs based on the comparative fit index [CFI; [59]] and the root mean square error of approximation [RMSEA; [39]] and compare model fits with the Akaike Information Criterion [AIC; [60]] and the likelihood ratio test. Following the recommendations by Browne and Cudeck [58] and Hu and Bentler [61], we consider CFI values > 0.90 and RMSEA values < 0.08 to indicate acceptable model fit and CFI values > 0.95 and RMSEA values < 0.06 to indicate good model fit. AIC differences ≥ 10 will be interpreted to indicate a substantial advantage in relative model fit in direct model comparisons [62]. We will assess the statistical significance of model parameters with the two-sided critical ratio test. To our knowledge, this is the first study to systematically monitor and evaluate long-term app usage in a German sample of older adults. We therefore lack the necessary prior knowledge to determine exclusion criteria for app usage and engagement with the tablet. Thus, a blinded analyst [63] will screen study partners’ app usage after the data is collected to propose appropriate criteria. The blinded analyst will not have access to the outcome variables, protecting the confirmatory nature of the statistical inference.

Outlier values in measured variables (assessments) will be removed if they exceed ± 3 SD of the sample mean of their respective study arm and study time point. In addition, we will exclude individuals whose responses appear random (e.g., identical responses to all questions). Physical activity data will only be included if the individual has worn the sensor for at least 4 days.

The type of missing data will be assessed and accounted for using the full information maximum likelihood method in all statistical models. If data are found to be Not Missing at Random, this will be addressed appropriately to minimize potential bias and stated as a limitation.

## Methods: monitoring

### Data monitoring committee

A study management group (SMG) was created with representatives of all field-relevant work packages including the project coordinators, statisticians, data managers, app administrators, and assessors. The SMG meets biweekly to supervise the incoming data flow and discuss upcoming problems in the data collection.

### Trial monitoring

There are no regularly scheduled Ethics Committee meetings, as the trial is considered a low-risk intervention. Eventual changes to the protocol in rare cases are initially discussed within the SMG and in cases also with the consortium. In some cases, advisory board members are also involved. If needed, issues and needs for (minor) adjustments are communicated to the ethics committee and the German Clinical Trials Register via an amendment.

### Harms and participant retention

All potential adverse events are systematically recorded by the project coordination. An adverse event is defined as any period of illness, hospitalization, or falls, regardless of the suspected cause. If clarification is required, medical questions will be forwarded to a study doctor. These reports are then reviewed by an independent safety monitor. SPs receive a tablet when they enter the survey, which they keep after study completion as an incentive. All study participants also receive individual feedback on selected measured areas. If an SP deviates from the study protocol or discontinues the study, the reasons for this are strictly documented using a specifically designed study management tool that also serves, for example, for documenting the screening procedure.

## Ethics

### Research ethics approval

SMART-AGE as a randomized controlled trial has pre-registered its design and hypotheses in the Open Science Framework (OSF) prior to study enrollment (10.17605/OSF.IO/YQEBW, 2023–04–28) and has also registered the study in the German Clinical Trials Register (https://drks.de/search/de/trial/DRKS00034316, 2024–05–29). While the OSF registration of the SMART-AGE trial was published prior to data collection, the trial registration in Germany was completed retrospectively due to internal administrative delays. All procedures performed in studies of SMART-AGE involving human participants are in accordance with the ethical standards of the 1964 Helsinki declaration and its later amendments. Approval for the study was received from the Institutional Ethics Review Board of Heidelberg University’s Medical Faculty (No. S-672/2022). Study partner enrollment is currently ongoing.

### Protocol amendments

Any changes made with relevance for SPs are communicated to them either through the SMART-AGE message service via tablet or during a HV. In addition, a weekly staff meeting is held with the project coordinators, the assessors, the hotline staff, and, if necessary, other members such as the app administrators to discuss upcoming questions or adverse events.

### Consent or assent

All participants give written informed consent after being provided with comprehensive study information and the opportunity to ask the assessor questions. Participants have the right to withdraw from the study at any time without any negative consequences.

### Confidentiality

All procedures undertaken in SMART-AGE are in full accordance with the strict German and European data security guidelines.^2^

### Ancillary and post-trial care

All study participants are formally disbanded and offered the possibility to report back any occurrences that might happen after having left the study and might be connected to the study and its intervention.

## Discussion

Robust evidence on the use of digital technology as a prevention means to strengthen multiple resources important to maintain QoL in old age is lacking. In terms of strengths, SMART-AGE first aims to improve this situation based on a complex trial design and a multimodal intervention by means of four interconnected apps. Target areas include enhancing social participation, increasing physical activity, and boosting health awareness, as well as the intensive collection of feedback, while using the respective apps.

Second, the exploitation of expected data including user-centered feedback and objective usage information may hold the potential for important insights. For example, effects observed at the primary outcome level (loneliness and social support, motor capacity and performance, health-related self-awareness and health control beliefs, technology acceptance and competence) can be linked with the objective app usage patterns and user feedback. This enables a deeper understanding of why and how the interventions work or do not work, and for whom.

Third, SMART-AGE also follows a multilevel approach of self-report data, performance-based data, and tracking data for assessing primary outcome domains. Therefore, possible differential effects of interventions on subjective versus objective assessment levels can be tested.

Fourth, SMART-AGE allows for examining short-term (3 months) and medium/long-term (6 months) effects of app use. Given the short cycles of technology and product innovation, we posit that 6 months is a sufficient time period for robust effect testing. For example, SMART-AGE’s digital appliances do not use speech control features, but it can be expected that speech control and speech assistance will much expand also in the area of digital preventive tools, e.g. [64].

Fifth, note that the SMART-AGE’s main study as described in this study protocol will be enriched by three in-depth sub-studies. First, we are utilizing 1-week Ecological Momentary Assessments (EMA) in subsamples (15% of each study arm) of those who have completed the main study to gain deeper insights into the contextual dynamics of activity and inactivity concerning technology usage, social participation, physical performance, and life space dynamics. Besides its short-term variability and short-term causal analysis potential, EMA data will also be used for additional exploratory outcome measurement. For example, EMA data extend the primary outcome of social engagement by objective data on time being alone or with others. Second, a qualitative sub-study of *N* = 20 study partners generated from the full intervention arm will delve into the ethical dimensions surrounding the utilization of digital technologies in older adults. For example, given our basic concepts of self-regulation versus co-regulation, this in-depth study aims to unfold in which app use may enhance or depress the experience of autonomy. Third, we conduct a qualitative study (*N* = 12) in the community with a lowered socio-economic background, in which we offer solely the smartVERNETZT app to cases much more diverse compared to the main intervention study. By this means, we hope to gain at least beginning evidence on how one of our apps may work in a more diverse population.

Still, a number of limitations should be acknowledged. First, SMART-AGE focuses on short to medium-term effects, hence outcomes beyond 6 months are not assessed. This limits the ability to draw conclusions about the sustainability of behavioral changes, which may require longer observation periods. Second, the different study arms require varying numbers of home visits, which could introduce a bias. For counterbalancing, about 25% of visits are replaced with video calls to examine how this difference might affect study partner engagement. It could, however, be that this social input and balancing might have been too low-dose in principle. Third, we expect sample selectivity and thus the bandwidth of generalization will be constrained. Fourth, for practical reasons, participants needed prior experience with a PC or tablet, which may limit generalizability. Finally, cultural and ethnic diversity was not specifically considered in the study design. As a result, the generalizability of the findings to non-German-speaking populations or other cultural contexts may be limited.

## Trial status

Protocol Version number and date: Version 1, 5. July 2024, Version 2, 28. July 2025, Version 3, 30.09.2025.

Date recruitment began: 9. May 2023.

End date of recruitment: 31. August 2024.

## Supplementary Information

Supplementary Material 1. 

Supplementary Material 2.

Supplementary Material 3.

Supplementary Material 4. 

Supplementary Material 5.

## Data Availability

The cleaned and pseudonymized SMART-AGE dataset will be made available for further research after the trial concludes and the main publications have been released. Access will be granted upon review and approval of a statistical analysis plan and will be limited to the specific variables outlined in that plan.

## Abbreviations

KOKU: Keep On Keep Up
IG: Intervention group
CG: Control group
HV: Home visit
QoL: Health-related quality of life
SMART-AGE: Smart Aging in Community Contexts: Testing Intelligent Assistive Systems for Self-regulation and Co-regulation under Real-Life Conditions
SP: Study partners
PRISM: Personal Reminder Information and Social Management System

## Footnotes

1 Given the now legally established retirement age of 67 years of age, the German Federal Statistical Office has changed its old age threshold to the age of 67 years. Therefore, we are using this age threshold to follow Germany’s current official statistical practice and avoid to sample those still in the workforce.

2 The date security concept containing all details of data handling and confidentially measures is available on request.
