# Supplementary material for: Testing a digitally administered intervention to increase social participation, physical fitness, and health awareness among healthy older adults by means of tablet-based app use: study protocol of the SMART-AGE randomized controlled trial
Source: Trials. 2026 Mar 21;27:285. doi: 10.1186/s13063-026-09641-3 (PMC13063763; doi:10.1186/s13063-026-09641-3)
Supplement: Supplementary file 1 — Supplementary Material 1. [file 13063_2026_9641_MOESM1_ESM.docx]

**Appendix**

The appendix presents the SMART-AGE Apps (A-1) and provides a comprehensive list of additional assessments, encompassing all measures related to secondary and exploratory hypotheses, as well as demographic evaluations that describe the sample (A-2).

**A1: SMART-AGE Apps**

**A-1a: smartVERNETZT**

Example of a smartVERNETZT screen: Overview over access to news (left side) about local events and concise articles covering topics such as social inclusion, physical activity, health, and technology, as well as public weather reports (right side). By tapping on 'Aktivitäten' (translated as 'Activities') in the top left corner, a menu with different features is displayed: Home, Email, Calendar, Video Chat, Entertainment, Learning, Health, Life and Daily Routine, Culture, Internet, and Contact.


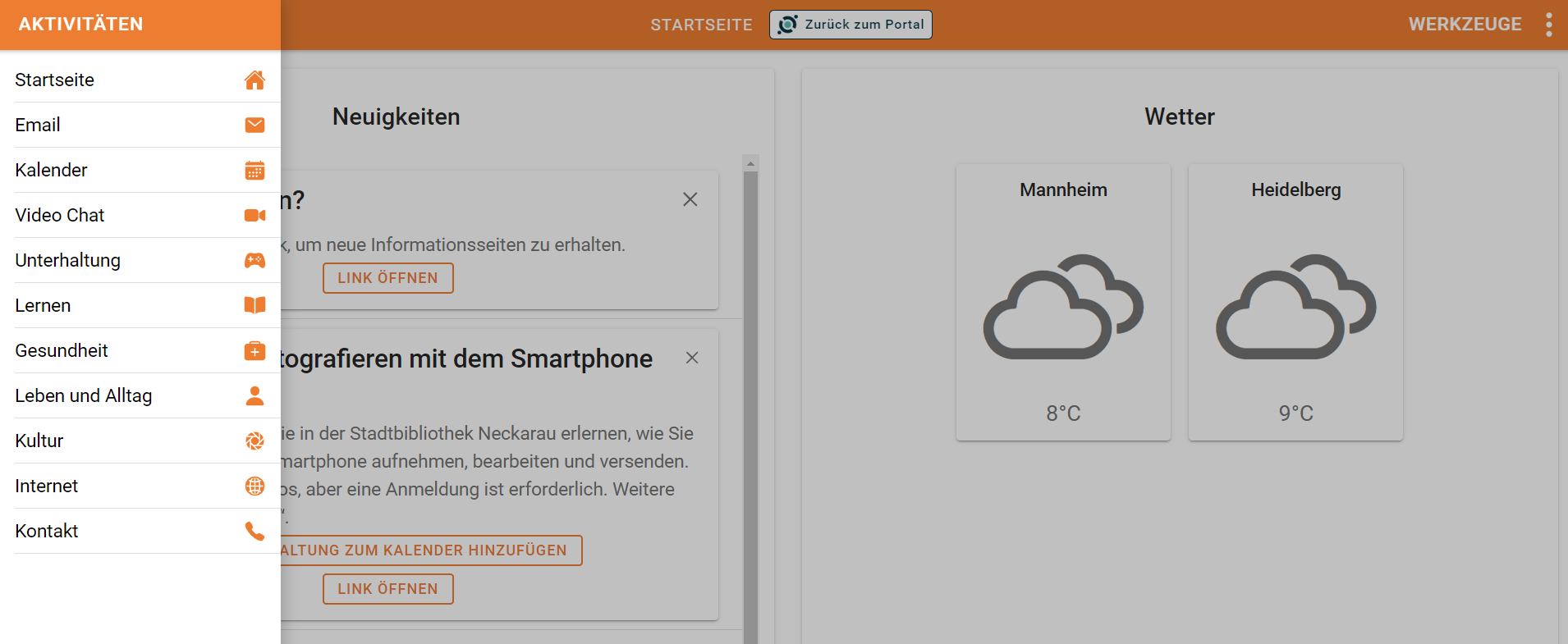


**A-1b: KOKU**

Four KOKU screens: 'Today's Exercises' (yellow), which shows the exercises planned for today; 'Your Progress' (green), indicating how many exercises have been completed in the weekly program; 'Games' (blue), including 4 different games to improve health and safety in the home; and 'Exercises' (red), offering a variety of exercises to choose from.


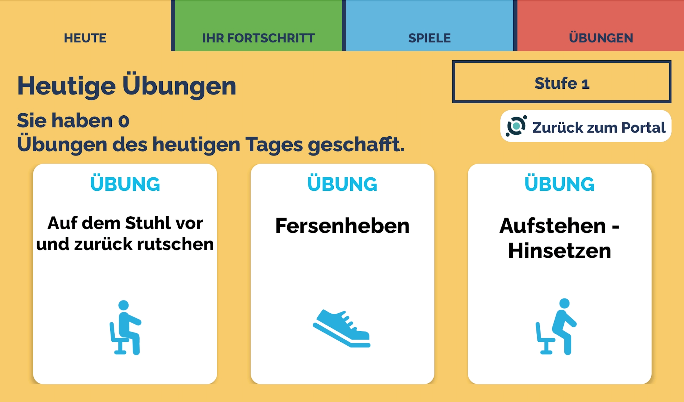

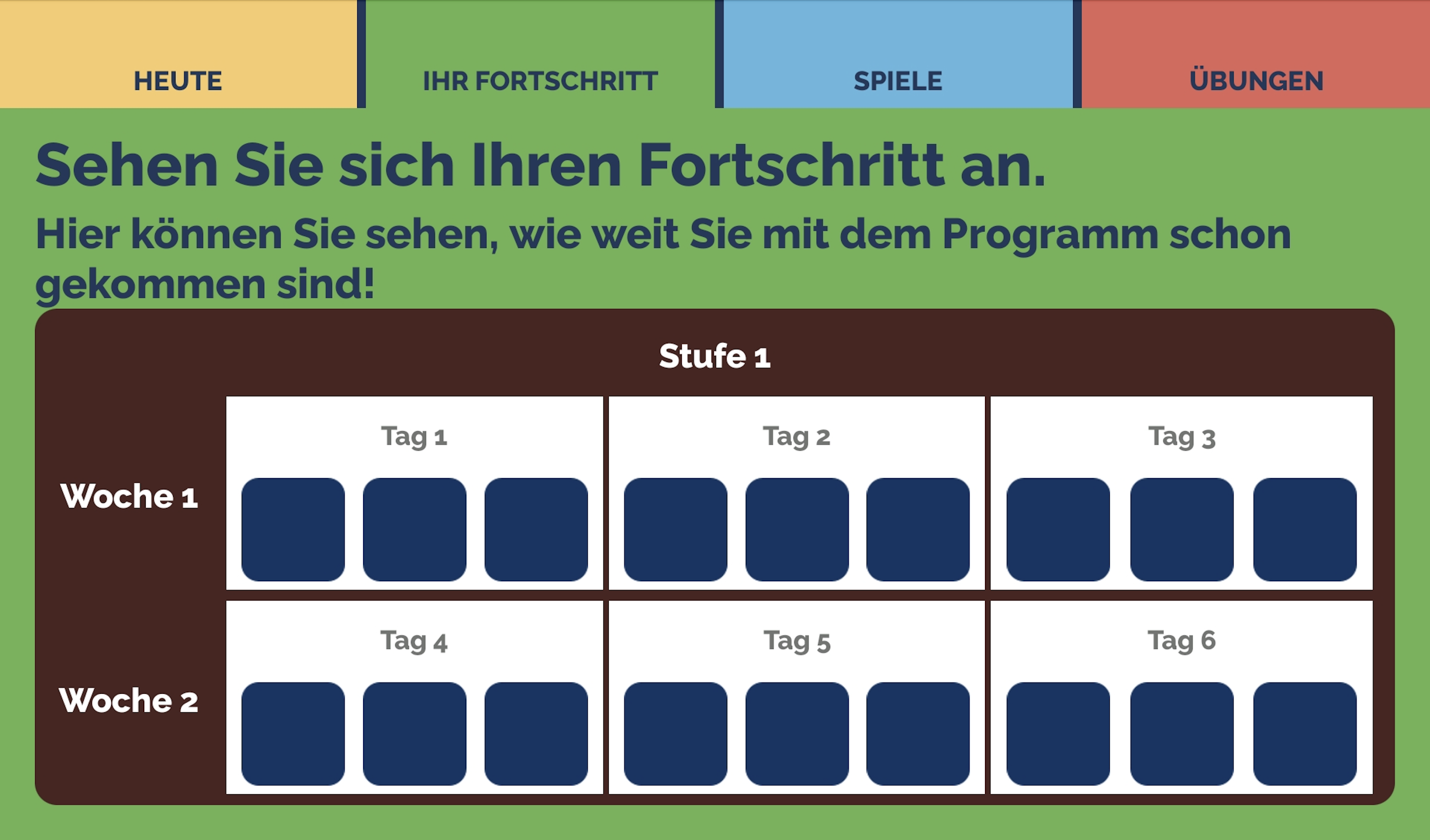

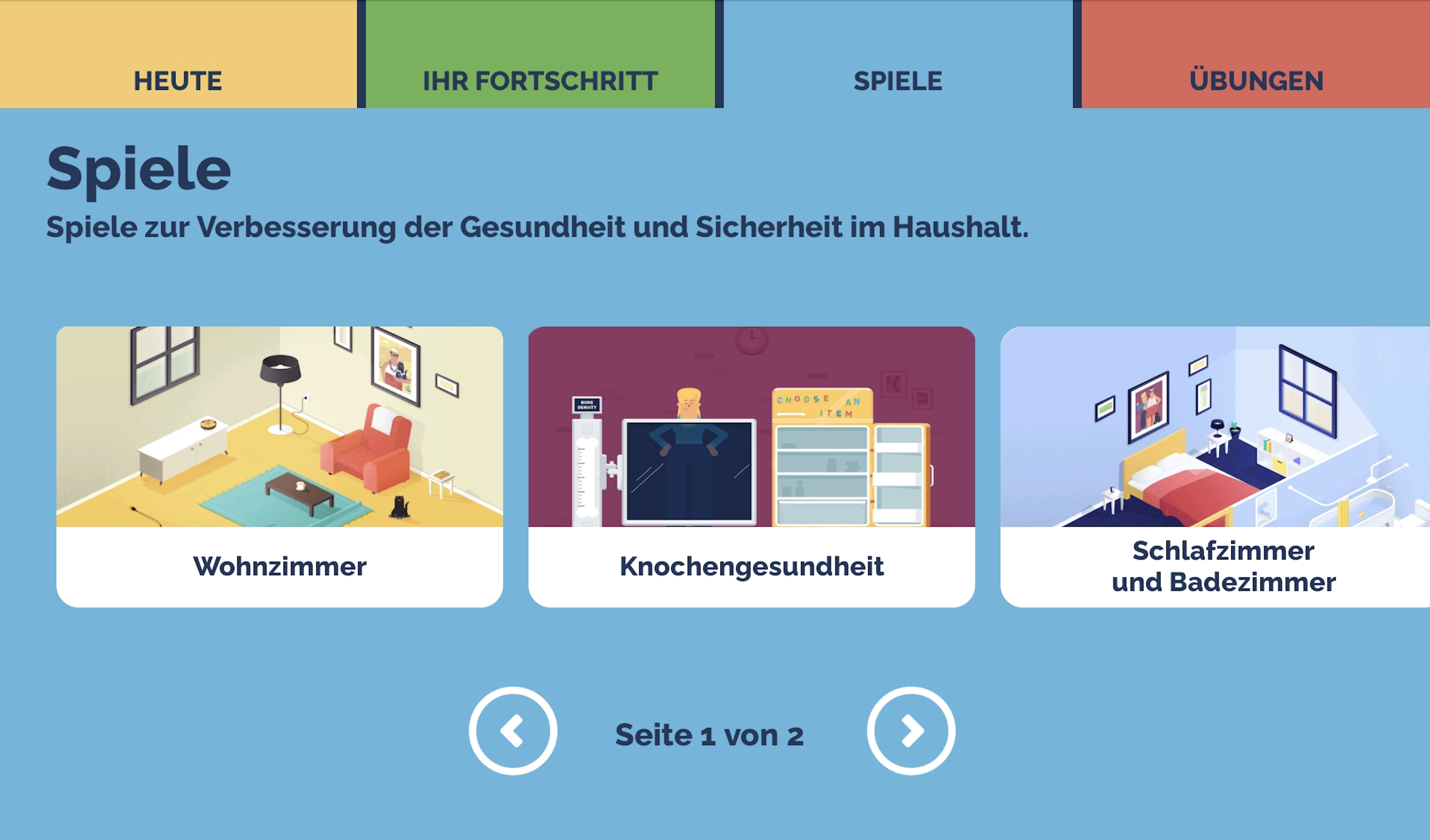

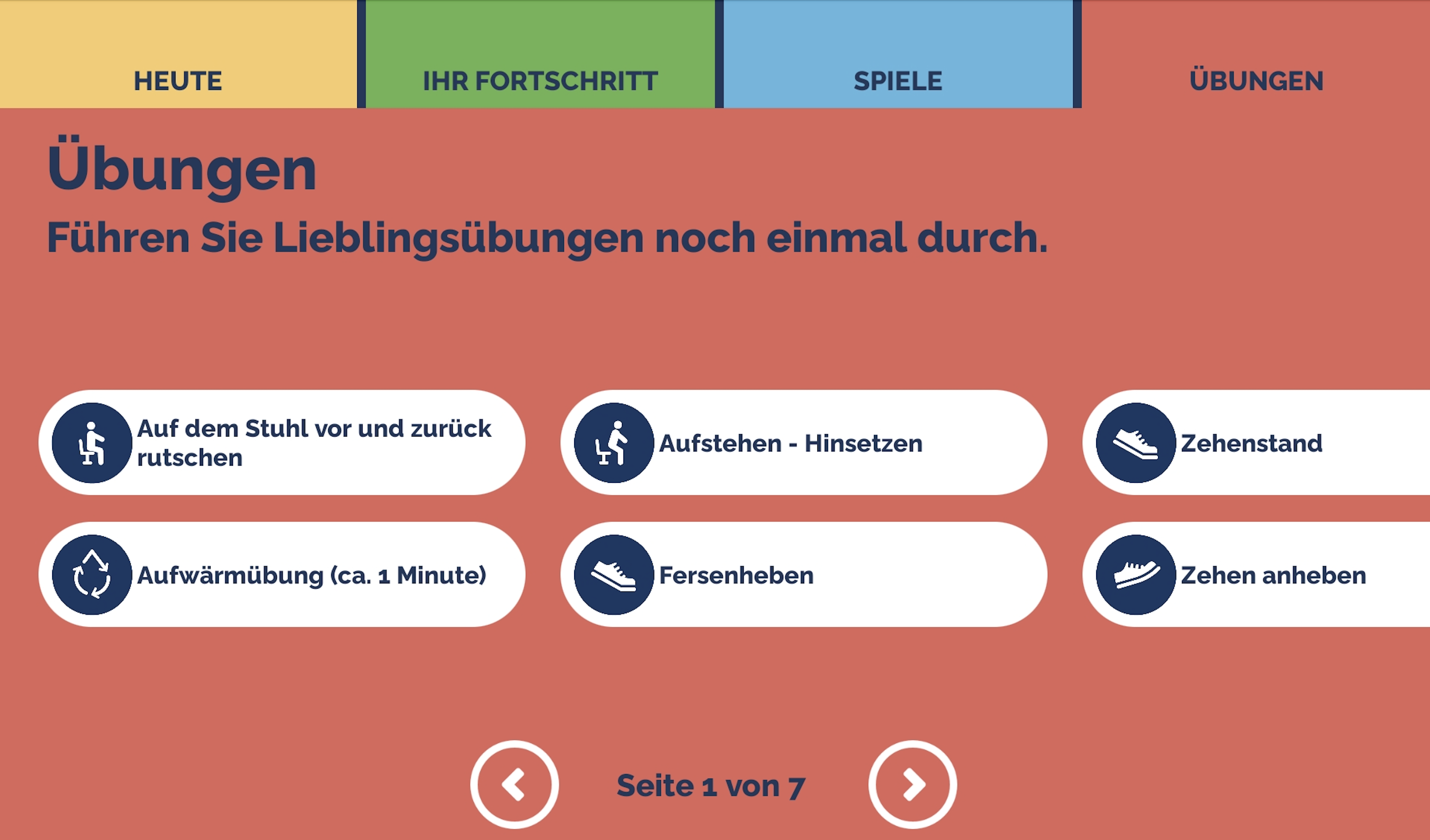


**A-1c: smartIMPULS**

Example of smartIMPULS screen: displaying how many questions have been answered and how many hints are available. On the left side, there are 5 options that can be selected: Home, Questions, Hints, Answers, and Profile.


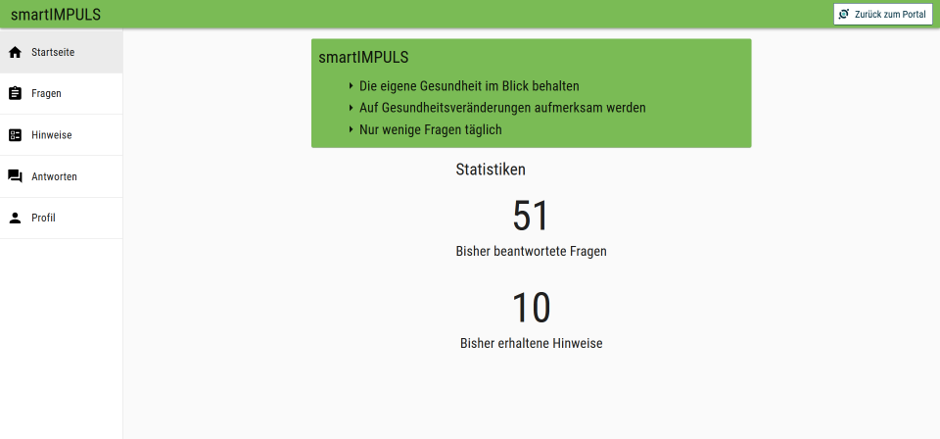


**A-1d: smartFEEDBACK**

Example of smartFEEDBACK screen: The red circled 3 indicates that there are 3 questions to answer. Here, users can answer questions sent by the research team. They can also specify the priority of their response and select an appropriate mood using a smiley. On the left side, there are additional options: Message, Sent, History, and Tip, which users can select.


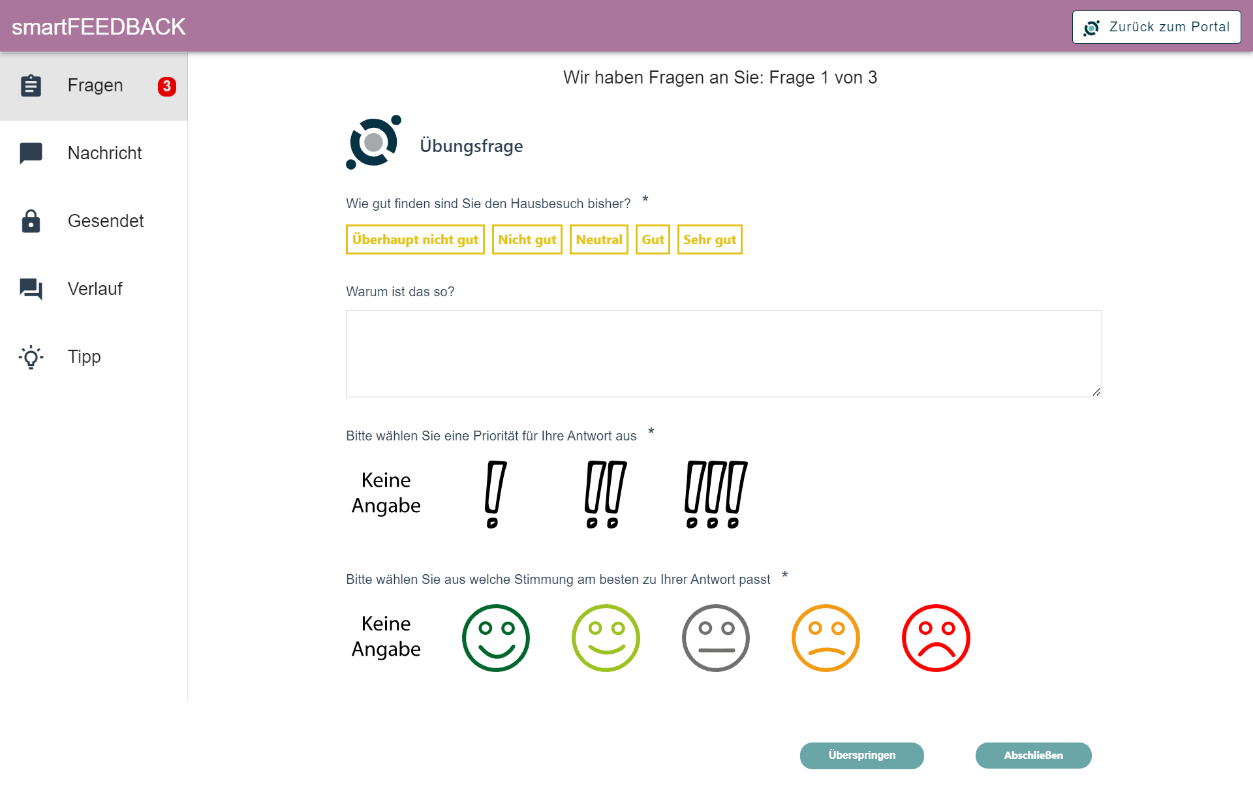


A

(a) Questions

(b) Message

(c) Sent

**A2: Assessments**

**A-2a: Primary Assessments**

| **Assessments to test the a priori hypotheses** | | |
| --- | --- | --- |
| **Outcome domains** | **Measurement** | **Source** |
| Health-related self-awareness, control beliefs, and self-efficacy | Health Locus of Control Scale | [1,2] |
|  | Health Literacy | [3] |
|  | Adapted Health Consciousness Scale | [4] |
|  | Domain-specific health awareness | [5] |
|  | Domain-specific health behavior routines | self-generated questionnaire |
|  | Self-efficacy | [5] |
|  | IT Self-Efficacy | self-generated questionnaire based on existing literature [6] |
| Motor capacity and performance | Timed Up-and-Go Test | [7] |
|  | movement-sensor based physical activity (e.g., steps per day) using Axivity AX6 sensors |  |
| Loneliness and social support | UCLA Loneliness Scale | [8] |
|  | Lubben Social Network Scale | [9] |
|  | Interpersonal Support Evaluation List | [10] |
| Technology acceptance, competence, and usage | Attitude Towards IT | self-generated questionnaire based on existing literature [11–14] |
|  | Perceived Usefulness and Perceived Ease of Use | self-generated questionnaire based on existing literature [15] |
|  | Intention to (continue) use | self-generated questionnaire based on existing literature [16] |
|  | Perceived Enjoyment | self-generated questionnaire based on existing literature [17] |
|  | Computer Anxiety | self-generated questionnaire based on existing literature [18] |
|  | Technostress | self-generated questionnaire based on existing literature [19] |
|  | End User Frustration | self-generated questionnaire based on existing literature [20] |
|  | Habitual behavior | self-generated questionnaire based on existing literature [21] |

**A-2b: Secondary Assessments**

| **Assessments to test the exploratory hypotheses** | | |
| --- | --- | --- |
| **Constructs** | **Measurement** | **Source** |
| Wellbeing | Satisfaction with life scale | [22] |
| Health Belief | Health Belief Model | self-generated questionnaire based on existing literature [23] |
| Quality of Life | WHOQOL-BREF | [24,25] |
| Health Literacy | E-Health Literacy | [26] |
| Usability Score von smartIMPULS | System Usability Scale (SUS) | [27] |
| Fall-related self-efficacy | SHORT FES-I | [28] |
| Spatial mobility | Life-Space Assessment | [29] |
| Motor function | gait speed | self-generated questionnaire based on existing literature [30,31] |
| Subjective memory | subjective memory | [32] |
| Depression | PHQ-8 | [33,34] |
| Digit Span Forward; Digit Span backward, Lexical Decision Task; Hagener Matrizen Test | cognitive exercises lab.js | [35–37] |
| Color Discrimination Task; BEFKI-GC; Location-Color Binding Task | cognitive exercises lab.js | [38–40] |
| "Verbale Flüssigkeit: Kategorie Tiere"[verbal fluency : category animals] (CERAD, Welsh et al., 1994); "Wortliste Lernen" [word list learning] (CERAD, Welsh et al., 1994) ; 3. Symbolsuche (WAIS-IV; Wechsler, 2008); "Wortliste Abrufen"[word list recall] (CERAD, Welsh et al., 1994), "Wortliste Wiedererkennen" [word list recognition] (CERAD, Welsh et al., 1994), "Trail Making Test A und B" (Reitan, 1956) | cognitive measures | [41–43] |
| Attention Tasks | Attention Tasks | Sources Attention Tasks (Implemented outside of RedCap) [36,40,44,45] |
| sense of community | sense of community | [46] |
| sense of community | digital networking with the neighborhood | [47] |
| Mobility | mobility | [48] |
| Housing importance | Housing importance "MOH" | [49] |
| Control Belief | housing-related control beliefs "HCQ" | [49,50] |
| Barriers in the home area | Housing Enabler | [51] |
| Personality | Big Five Personality | [52] |
| Awareness of age-related Change | Awareness of age-related Change | [53] |
| Social activities | List of leisure activities | self-generated questionnaire based on existing literature [54] |
| Life Engagement | Life Engagement Test | [55] |
| Experienced obsolescence | Experienced obsolescence | [56] |
| Mobile device proficiency | Mobile Device Proficiency Questionnaire | [57] |
| Internet-Self-Efficacy | Internet-Self-Efficacy | [58,59] |
| Stereotype Endorsement | Stereotype Endorsement | [60] |
| Network Stereotype Endorsement | Perceived IT-Based Stereotype Endorsement | [61] |
| Techno-Induced Overload | Techno-Induced Overload | [62,63] |
| Perceived Achievement | Feelings of Achievement | [64] |
| Sociodemographic characteristics | Gender, Citizenships, Household members, Family members, Marital status, Housing, Long-term care insurance, Children, Grandchildren, Subjective standard of living, Community, Income, Education, Job, Retirement | self-generated questionnaire based on existing literature [54] |
| Wellbeing | Satisfaction with life scale | [22] |
| Comorbidity | Functional Comorbidity Index, | [65] |
| Technology usage | Media use/frequency of technology use | self-generated questionnaire, inspired by [66] |
| Technology usage | Frequency and type of Internet use | self-generated questionnaire, inspired by [54] |
| Technology Biography | Technology Biography | [67,68] |
| Emotion and Attitude from network | Emotion/Attitude toward IT from Network | [11–14] |
| Network End User Frustration | Perceived End User Frustration | [20] |
| cognitive screening | 6CIT | [69] |

# References

[1] Wallston BS, Wallston KA, Kaplan GD., Maides SA. Development and Validation of the Health Locus of Control (HLC) Scale. J Consult Clin Psychol 1976;44:580–5. https://doi.org/10.1037/0022-006X.44.4.580.

[2] Roski R, Schikorra S. Informations- und Medienverhalten von Versicherten und Patienten – Eine Segmentierung von Barmer Versicherten. In: Roski R, editor. Zielgruppengerechte Gesundheitskommunikation, Wiesbaden: VS Verlag für Sozialwissenschaften; 2009, p. 107–30. https://doi.org/10.1007/978-3-531-91476-3_5.

[3] Konopik N, Kaspar R, Penger S, Oswald F, Himmelsbach I. Advancing health literacy measurement in old age. Health Promot Int 2021;36:1310–23. https://doi.org/10.1093/heapro/daaa137.

[4] Marsall M, Engelmann G, Skoda E-M, Teufel M, Bäuerle A. Validation and Test of Measurement Invariance of the Adapted Health Consciousness Scale (HCS-G). Int J Environ Res Public Health 2021;18:6044. https://doi.org/10.3390/ijerph18116044.

[5] Jerusalem M, Schwarzer R. SWE. Skala zur Allgemeinen Selbstwirksamkeitserwartung [Verfahrensdokumentation, Autorenbeschreibung und Fragebogen]. Trier ZPID 2003. https://doi.org/10.23668/psycharchives.4515.

[6] Correia J, Compeau D, Thatcher J. Implications of Technological Progress for the Measurement of Technology Acceptance Variables: The Case of Self-efficacy. DIGIT 2016 Proc 2016.

[7] Podsiadlo D, Richardson S. The Timed “Up & Go”: A Test of Basic Functional Mobility for Frail Elderly Persons. J Am Geriatr Soc 1991;39:142–8. https://doi.org/10.1111/j.1532-5415.1991.tb01616.x.

[8] Döring N, Bortz J. Psychometrische Einsamkeitsforschung: Deutsche Neukonstruktion der UCLA Loneliness Scale. [Psychometric research on loneliness: A new German version of the University of California at Los Angeles (UCLA) Loneliness Scale.]. Diagnostica 1993;39:224–39.

[9] Lubben J, Blozik E, Gillmann G, Iliffe S, von Renteln Kruse W, Beck JC, et al. Performance of an Abbreviated Version of the Lubben Social Network Scale Among Three European Community-Dwelling Older Adult Populations. The Gerontologist 2006;46:503–13. https://doi.org/10.1093/geront/46.4.503.

[10] Merz EL, Roesch SC, Malcarne VL, Penedo FJ, Llabre MM, Weitzman OB, et al. Validation of Interpersonal Support Evaluation List-12 (ISEL-12) scores among English- and Spanish-Speaking Hispanics/Latinos from the HCHS/SOL Sociocultural Ancillary Study. Psychol Assess 2014;26:384–94. https://doi.org/10.1037/a0035248.

[11] Boser RA, Palmer JD, Daugherty MK. Students Attitudes Toward Technology in Selected Technology Education Programs. J Technol Educ 1998;10.

[12] Crites SL, Fabrigar LR, Petty RE. Measuring the Affective and Cognitive Properties of Attitudes: Conceptual and Methodological Issues. Pers Soc Psychol Bull 1994;20:619–34. https://doi.org/10.1177/0146167294206001.

[13] Yang H, Yoo Y. It’s all about attitude: revisiting the technology acceptance model. Decis Support Syst 2004;38:19–31. https://doi.org/10.1016/S0167-9236(03)00062-9.

[14] Zhang P. The Affective Response Model: A Theoretical Framework of Affective Concepts and Their Relationships in the ICT Context. MIS Q 2013;37:247–74. https://doi.org/10.25300/MISQ/2013/37.1.11.

[15] Davis FD. Perceived Usefulness, Perceived Ease of Use, and User Acceptance of Information Technology. MIS Q 1989;13:319–40. https://doi.org/10.2307/249008.

[16] Bhattacherjee, Anol, Premkumar, G. Understanding Changes in Belief and Attitude toward Information Technology Usage: A Theoretical Model and Longitudinal Test. MIS Q 2004;28:229–54. https://doi.org/10.2307/25148634.

[17] Van der Heijden H. User Acceptance of Hedonic Information Systems. MIS Q 2004;28:695–704. https://doi.org/10.2307/25148660.

[18] Thatcher JB, Perrewé PL. An Empirical Examination of Individual Traits as Antecedents to Computer Anxiety and Computer Self-Efficacy. MIS Q 2002;26:381–96. https://doi.org/10.2307/4132314.

[19] Ayyagari R. Impact of Information Overload and Task-technology Fit on Technostress. SAIS 2012 Proc 2012;4.

[20] Peters LH, O’Connor EJ, Rudolf CJ. The behavioral and affective consequences of performance-relevant situational variables. Organ Behav Hum Perform 1980;25:79–96. https://doi.org/10.1016/0030-5073(80)90026-4.

[21] Busch PA, Hausvik GI, Ropstad OK, Pettersen D. Smartphone usage among older adults. Comput Hum Behav 2021;121:106783. https://doi.org/10.1016/j.chb.2021.106783.

[22] Diener E, Emmons RA, Larsen RJ, Griffin S. The Satisfaction With Life Scale. J Pers Assess 1985;49:71–5. https://doi.org/10.1207/s15327752jpa4901_13.

[23] Jones CJ, Smith H, Llewellyn C. Evaluating the effectiveness of health belief model interventions in improving adherence: a systematic review. Health Psychol Rev 2014;8:253–69. https://doi.org/10.1080/17437199.2013.802623.

[24] Skevington SM, Lotfy M, O’Connell KA. The World Health Organization’s WHOQOL-BREF quality of life assessment: psychometric properties and results of the international field trial. A report from the WHOQOL group. Qual Life Res 2004;13:299–310.

[25] World Health Organization. Programme on mental health : WHOQOL user manual 1998. https://iris.who.int/handle/10665/77932 (accessed July 1, 2024).

[26] Soellner R, Huber S, Reder M. The Concept of eHealth Literacy and its Measurement: German Translation of the eHEALS. J Media Psychol Theor Methods Appl 2014;26:29–38. https://doi.org/10.1027/1864-1105/a000104.

[27] Brooke J. SUS-A quick and dirty usability scale. In: Jordan PW, Thomas B, McClelland IL, Weerdmeester B, editors. Usability Eval. Ind. 1st ed., London: CRC Press; 1996, p. 189–94.

[28] Kempen GIJM, Yardley L, Van Haastregt JCM, Zijlstra GAR, Beyer N, Hauer K, et al. The Short FES-I: a shortened version of the falls efficacy scale-international to assess fear of falling. Age Ageing 2008;37:45–50. https://doi.org/10.1093/ageing/afm157.

[29] Peel C, Baker PS, Roth DL, Brown CJ, Bodner EV, Allman RM. Assessing Mobility in Older Adults: The UAB Study of Aging Life-Space Assessment. Phys Ther 2005;85:1008–19. https://doi.org/10.1093/ptj/85.10.1008.

[30] Freire AN, Guerra RO, Alvarado B, Guralnik JM, Zunzunegui MV. Validity and Reliability of the Short Physical Performance Battery in Two Diverse Older Adult Populations in Quebec and Brazil. J Aging Health 2012;24:863–78. https://doi.org/10.1177/0898264312438551.

[31] Kim H, Park I, Lee H joo, Lee O. The reliability and validity of gait speed with different walking pace and distances against general health, physical function, and chronic disease in aged adults. J Exerc Nutr Biochem 2016;20:46–50. https://doi.org/10.20463/jenb.2016.09.20.3.7.

[32] Hülür G, Hertzog C, Pearman AM, Gerstorf D. Correlates and Moderators of Change in Subjective Memory and Memory Performance: Findings from the Health and Retirement Study. Gerontology 2015;61:232–40. https://doi.org/10.1159/000369010.

[33] Shin C, Lee S-H, Han K-M, Yoon H-K, Han C. Comparison of the Usefulness of the PHQ-8 and PHQ-9 for Screening for Major Depressive Disorder: Analysis of Psychiatric Outpatient Data. Psychiatry Investig 2019;16:300–5. https://doi.org/10.30773/pi.2019.02.01.

[34] Kroenke K, Strine TW, Spitzer RL, Williams JBW, Berry JT, Mokdad AH. The PHQ-8 as a measure of current depression in the general population. J Affect Disord 2009;114:163–73. https://doi.org/10.1016/j.jad.2008.06.026.

[35] Heydasch T, Haubrich J, Renner K-H. Die Kurzform des Hagener Matrizen-Tests (HMT-S): Ein 6-Item Intelligenztest zum schlussfolgernden Denken [The short version of the Hagen Matrices Test (HMT-S): 6-item induction intelligence test]. Methods Data Anal 2013;7:183–208. https://doi.org/10.12758/mda.2013.011.

[36] Ratcliff R, Gomez P, McKoon G. A Diffusion Model Account of the Lexical Decision Task. Psychol Rev 2004;111:159–82. https://doi.org/10.1037/0033-295X.111.1.159.

[37] Wechsler D. WISC-IV: Wechsler intelligence scale for children: Technical and interpretive manual. Psychological Corporation; 2004.

[38] Oberauer K, Lin H-Y. An Interference Model of Visual Working Memory. Psychol Rev 2017;124:21–59. https://doi.org/10.1037/rev0000044.

[39] Schipolowski S, Wilhelm O, Schroeders U, Kovaleva A, Kemper CJ, Rammstedt B. Eine kurze Skala zur Messung kristalliner Intelligenz: Die Kurzskala gc des Berliner Tests zur Erfassung Fluider und Kristalliner Intelligenz (BEFKI GC-K). Mannh GESIS - Leibniz-Inst Für Sozialwissenschaften 2014.

[40] Voss A, Rothermund K, Voss J. Interpreting the parameters of the diffusion model: An empirical validation. Mem Cognit 2004;32:1206–20. https://doi.org/10.3758/BF03196893.

[41] Bowie CR, Harvey PD. Administration and interpretation of the Trail Making Test. Nat Protoc 2006;1:2277–81. https://doi.org/10.1038/nprot.2006.390.

[42] Wechsler D. Wechsler adult intelligence scale: WAIS-IV; technical and interpretive manual. Pearson; 2008.

[43] Welsh KA, Butters N, Mohs RC, Beekly D, Edland S, Fillenbaum G, et al. The Consortium to Establish a Registry for Alzheimer’s Disease (CERAD). Part V. A normative study of the neuropsychological battery. Neurology 1994;44:609–14. https://doi.org/10.1212/WNL.44.4.609.

[44] Oberauer K, Süß H-M, Schulze R, Wilhelm O, Wittmann WW. Working memory capacity — facets of a cognitive ability construct. Personal Individ Differ 2000;29:1017–45. https://doi.org/10.1016/S0191-8869(99)00251-2.

[45] Stepankova H, Lukavsky J, Buschkuehl M, Kopecek M, Ripova D, Jaeggi SM. The Malleability of Working Memory and Visuospatial Skills: A Randomized Controlled Study in Older Adults. Dev Psychol 2014;50:1049–59. https://doi.org/10.1037/a0034913.

[46] Peterson NA, Speer PW, McMillan DW. Validation of A brief sense of community scale: Confirmation of the principal theory of sense of community. J Community Psychol 2008;36:61–73. https://doi.org/10.1002/jcop.20217.

[47] Kurtenbach S, Küchler A, Rees Y. Digitalisierung und nachbarschaftlicher Zusammenhalt im ländlichen Raum: Ergebnisse einer Mixed-Methods-Untersuchung. Raumforsch Raumordn Spat Res Plan 2022;80:329–43. https://doi.org/10.14512/rur.108.

[48] Penger S, Oswald F. A New Measure of Mobility-Related Behavioral Flexibility and Routines in Old Age. GeroPsych 2017;30:153–63. https://doi.org/10.1024/1662-9647/a000176.

[49] Oswald F, Schilling O, Wahl H-W, Fänge A, Sixsmith J, Iwarsson S. Homeward bound: Introducing a four-domain model of perceived housing in very old age. J Environ Psychol 2006;26:187–201. https://doi.org/10.1016/j.jenvp.2006.07.002.

[50] Oswald F, Wahl H-W, Martin M, Mollenkopf H. Toward Measuring Proactivity in Person-Environment Transactions in Late Adulthood: The Housing-Related Control Beliefs Questionnaire. J Hous Elder 2003;17:135–52. https://doi.org/10.1300/J081v17n01_10.

[51] Norin L, Iwarsson S, Haak M, Slaug B. The Housing Enabler instrument: Assessing threats to reliability and validity. Br J Occup Ther 2019;82:48–59. https://doi.org/10.1177/0308022618782329.

[52] Rammstedt B, John OP. Kurzversion des Big Five Inventory (BFI-K): Entwicklung und Validierung eines ökonomischen Inventarszur Erfassung der fünf Faktoren der Persönlichkeit. Diagnostica 2005;51:195–206. https://doi.org/10.1026/0012-1924.51.4.195.

[53] Kaspar R, Gabrian M, Brothers A, Wahl H-W, Diehl M. Measuring Awareness of Age-Related Change: Development of a 10-Item Short Form for Use in Large-Scale Surveys. The Gerontologist 2019;59:e130–40. https://doi.org/10.1093/geront/gnx213.

[54] Vogel C, Klaus D, Wettstein M, Simonson J, Tesch-Römer C. German Ageing Survey (DEAS). In: Gu D, Dupre ME, editors. Encycl. Gerontol. Popul. Aging. 1st ed., Cham: Springer; 2021, p. 2152–60. https://doi.org/10.1007/978-3-030-22009-9_1115.

[55] Scheier MF, Wrosch C, Baum A, Cohen S, Martire LM, Matthews KA, et al. The Life Engagement Test: Assessing Purpose in Life. J Behav Med 2006;29:291–8. https://doi.org/10.1007/s10865-005-9044-1.

[56] Brandtstädter J, Wentura D, Schmitz U. Veränderungen der zeit- und zukunftsperspektive im übergang zum höheren alter: Quer- und längsschnittliche befunde. [Age-related changes in future time perspectives: Cross-sectional and longitudinal findings.]. Z Für Psychol Mit Z Für Angew Psychol 1997;205:377–95.

[57] Roque NA, Boot WR. A New Tool for Assessing Mobile Device Proficiency in Older Adults: The Mobile Device Proficiency Questionnaire. J Appl Gerontol 2018;37:131–56. https://doi.org/10.1177/0733464816642582.

[58] Jokisch MR, Schmidt LI, Doh M, Marquard M, Wahl H-W. The role of internet self-efficacy, innovativeness and technology avoidance in breadth of internet use: Comparing older technology experts and non-experts. Comput Hum Behav 2020;111:106408. https://doi.org/10.1016/j.chb.2020.106408.

[59] Schenk M, Scheiko L. Meinungsführer als Innovatoren und Frühe Übernehmer des Web 2.0. Media Perspekt 2011;9:423–31.

[60] Schmader T, Johns M, Forbes C. An integrated process model of stereotype threat effects on performance. Psychol Rev 2008;115:336–56. https://doi.org/10.1037/0033-295X.115.2.336.

[61] Rattan A, Savani K, Komarraju M, Morrison MM, Boggs C, Ambady N. Meta-lay theories of scientific potential drive underrepresented students’ sense of belonging to science, technology, engineering, and mathematics (STEM). J Pers Soc Psychol 2018;115:54–75. https://doi.org/10.1037/pspi0000130.

[62] Pethig F, Kroenung J, Noeltner M. A stigma power perspective on digital government service avoidance. Gov Inf Q 2021;38:101545. https://doi.org/10.1016/j.giq.2020.101545.

[63] Maier C, Laumer S, Eckhardt A. Information technology as daily stressor: pinning down the causes of burnout. J Bus Econ 2015;85:349–87. https://doi.org/10.1007/s11573-014-0759-8.

[64] Pekrun R, Goetz T, Frenzel AC, Barchfeld P, Perry RP. Measuring emotions in students’ learning and performance: The Achievement Emotions Questionnaire (AEQ). Contemp Educ Psychol 2011;36:36–48. https://doi.org/10.1016/j.cedpsych.2010.10.002.

[65] Groll DL, To T, Bombardier C, Wright JG. The development of a comorbidity index with physical function as the outcome. J Clin Epidemiol 2005;58:595–602. https://doi.org/10.1016/j.jclinepi.2004.10.018.

[66] Wagner M, Zank S, editors. Abschlussbericht: Lebensqualität und Wohlbefinden hochaltriger Menschen in NRW (Folgebefragung NRW80+). Köln: Universität zu Köln, Cologne Center for Ethics, Rights, Economics, and Social Sciences of Health (ceres); 2022.

[67] Claßen K. Zur Psychologie von Technikakzeptanz im höheren Lebensalter: Die Rolle von Technikgenerationen 2013. https://doi.org/10.11588/heidok.00014295.

[68] Mollenkopf H, Meyer S, Schulze E, Wurm S, Friesdorf W. Technik im Haushalt zur Unterstützung einer selbstbestimmten Lebensführung im Alter Das Forschungsprojekt „sentha” und erste Ergebnisse des Sozialwissenschaftlichen Teilprojekts. Zeitschrift für Gerontologie und Geriatrie 2000;33:155–68. https://doi.org/10.1007/s003910070056.

[69] Gale TM, Larner AJ. Six-Item Cognitive Impairment Test (6CIT). In: Larner AJ, editor. Cogn. Screen. Instrum. Pract. Approach. 2nd ed., Cham: Springer; 2017, p. 241–53. https://doi.org/10.1007/978-3-319-44775-9_11.
