# Supplementary material for: Testing a digitally administered intervention to increase social participation, physical fitness, and health awareness among healthy older adults by means of tablet-based app use: study protocol of the SMART-AGE randomized controlled trial
Source: Trials. 2026 Mar 21;27:285. doi: 10.1186/s13063-026-09641-3 (PMC13063763; doi:10.1186/s13063-026-09641-3)
Supplement: Supplementary file 3 — Supplementary Material 3. [file 13063_2026_9641_MOESM3_ESM.pdf]

MEDIZINISCHE
FAKULTÄT
HEIDELBERG

Ethikkommission der Med. Fak. HD | Alte Glockengießerei 11/1 | 69115 Heidelberg

Herrn Prof. Dr. Jürgen Bauer
Agaplesion Bethanien Krankenhaus Heidelberg
Geriatrisches Zentrum am Universitätsklinikum
Rohrbacher Straße 149
69126 Heidelberg

**Universität Heidelberg
Ethikkommission der
Med. Fakultät**

Alte Glockengießerei 11/1
69115 Heidelberg

Tel. +49 6221 56264-60 (Zentrale)
Fax +49 6221 56264-80
ethikkommission-I@med.uni-heidelberg.de

www.medizinische-fakultaet-hd.uni-heidelberg.de/einrichtungen/zentrale-einrichtungen/ethikkommission

Vorsitz:
Prof. Dr. med. Dr. h.c. Thomas Strowitzki

Stellv. Vorsitz:
Prof. Dr. med. David Czock
Dr. sc. hum. Anja Sander

Geschäftsleitung:
Dr. med. Verena Pfeilschifter

06.06.2024
ts-bw

CONFIRMATION

Reference No: **S-672/2022**

Title: **Smartes Altern im kommunalen Kontext: Untersuchung intelligenter Formen von Selbstregulation und Ko-Regulation unter Realbedingungen (SMART-AGE)**

Dear Mr. Professor Bauer,

we hereby confirm that the above-mentioned research project has been reviewed and approved by the Ethics Committee of the Medical Faculty of the University of Heidelberg. The approval of the Ethics Committee (Berufsrechtliche Beratung), dated to 26.03.2024, has been sent to you on 27.03.2024.

Kind regards

Digital unterschrieben
von Dr. Thomas Strowitzki
Datum: 2024.06.06
13:32:46 +02'00'

Prof. Dr. med. Dr. h.c. Thomas Strowitzki
Vorsitzender

Qualitätsmanagement ISO 9001:2015 399893 QM15

Informationssicherheit ISO/IEC 27001 399893 ISMS17

BIC SOLADEST600
IBAN DE64 6005 0101 7421 5004 29

MF HD

.
