# Supplementary material for: Testing a digitally administered intervention to increase social participation, physical fitness, and health awareness among healthy older adults by means of tablet-based app use: study protocol of the SMART-AGE randomized controlled trial
Source: Trials. 2026 Mar 21;27:285. doi: 10.1186/s13063-026-09641-3 (PMC13063763; doi:10.1186/s13063-026-09641-3)
Supplement: Supplementary file 4 — Supplementary Material 4. [file 13063_2026_9641_MOESM4_ESM.pdf]

Carl-Zeiss-Stiftung, Breitscheidstraße 10, 70174 Stuttgart

22nd of July 2024

Letter of Confirmation

To Whom it may concern,

This letter serves to confirm the multi-site research project

"Smart Aging in Community Contexts: Testing Intelligent Assistive Systems for Self-regulation and Co-regulation under Real-Life Conditions (SMART-AGE)"

Lukas Findeisen
Program Manager
+49 711 16221320
Lukas.findeisen@carl-zeiss-stiftung.de

addressed in a recently submitted Study Protocol at scientific journal TRIALS (TRLS-D-24-00911) received funding approval by the Carl-Zeiss-Stiftung on October 22, 2020. It is administered under the project number P2019-01-003.

Official project receiver is Heidelberg University, represented by its Network Aging Research as well as other university faculty institutes. External academic institutions such as the Universities of Mannheim and Mainz are also involved based on respective contracting. Funding is secured by the Carl-Zeiss-Stiftung from April 1, 2021, to March 31, 2026, with an amount of 4.5 Mio EUR. The funding was award within the Foundation's most competitive "Breakthroughs" ("Durchbrüche") funding program. Due to legal obligations by the federal state of Germany all legal funding documents must be in German language. However, the foundation hereby confirms the information communicated in this letter is a representation of the legally binding documents in German language.

SMART-AGE assesses framed as a randomized complex trial, whether and how a set of apps aimed to enhance social participation, health, and physical fitness in 67+ year-old individuals may impact on quality of life.

The Carl-Zeiss-Stiftung expects the project's goals and results to be made accessible to the public and to acknowledge the support of the Carl-Zeiss-Stiftung in all dissemination activities.

About Carl-Zeiss Stiftung

Founded in 1889 by scientist and entrepreneur Prof. Ernst Abbe, the Carl Zeiss Foundation is one of Germany's largest and oldest science-funding foundations. Its funding activities are financed from the dividends of its foundation companies, Carl Zeiss AG and SCHOTT AG, which have been in the inalienable

sole ownership of the Carl-Zeiss-Stiftung for over a hundred years. The Foundation provides funding for research and teaching in the STEM fields of science, technology, engineering, and mathematics, focusing on key topics that address current scientific, economic, and social challenges. The funding activities primarily target universities and scientific institutions in Baden-Württemberg, Rhineland-Palatinate, and Thuringia.

Yours sincerely

Stuttgart, 22.07.2024

Lukas Findeisen
Program Manager for Artificial Intelligence and Life Science Technologies

(Further information can be found on the homepage: https://www.carl-zeiss-stiftung.de/en/)
