# Supplementary material for: Testing a digitally administered intervention to increase social participation, physical fitness, and health awareness among healthy older adults by means of tablet-based app use: study protocol of the SMART-AGE randomized controlled trial
Source: Trials. 2026 Mar 21;27:285. doi: 10.1186/s13063-026-09641-3 (PMC13063763; doi:10.1186/s13063-026-09641-3)
Supplement: Supplementary file 5 — Supplementary Material 5. [file 13063_2026_9641_MOESM5_ESM.pdf]

Smart Aging in Community Contexts: Testing Intelligent Assistive Systems for Self-regulation and Co-regulation under Real-Life Conditions

Declaration of consent for participation in the SMART-AGE study

I have received and read the SMART-AGE information sheet and have been informed in detail and comprehensibly by Mr./Mrs. __________________ about the aim and the process of the study as well as about the risks. I had the opportunity to ask questions during the information session. All my questions were answered to my satisfaction. I had enough time to make my decision.

I, _________________________, _________________________
(surname) (first name)

born on ______________________,

voluntarily agree to participate in the SMART-AGE study. I have received a copy of the information leaflet and the declaration of consent.

I am aware that I will be randomly assigned to one of the three study groups after the first home visit. The study groups differ in terms of the number of home visits and the apps provided. It is not possible to change to a study group other than the one randomly assigned. I agree with this regulation.

I understand that I will not incur any costs by participating in the study and that it will run for a maximum of one year. In return for my participation, the tablet required for the study will become my property, provided that I have participated in the study for the entire study period.

I agree to the highly protected storage of my personal data (surname, first name, date of birth, address, telephone number) until the end of the SMART-AGE study (31.03.2026). This is necessary because participation in SMART-AGE requires repeated contact and associated home visits.

I will receive a copy of the declaration of consent.

As part of the survey, changes and possible indications of health problems can be noted on the basis of the results. I am free to decide whether these are discussed with me or not ("right to remain ignorant").

If I wish to be informed about this, I will be told how to proceed. I am usually advised to discuss this with my family doctor. The study staff will not contact my family doctor for this purpose. This is exclusively my right.

I have been informed and voluntarily agree that my data collected in the study, in particular information about my health and my ethnic origin, will be recorded in pseudonymized form and evaluated in anonymized form for the purpose described in the information leaflet.

The data collected in SMART-AGE includes all information that I provide via questionnaires about my health, my well-being and my attitude to technology, the results of cognitive and motor tests and the recording of my movement data using an activity sensor. Depending on the study group to which I am assigned, pseudonymized usage data of the apps used in SMART-AGE may also be collected, for example how often and for how long I have accessed a particular app or what information I have viewed in the app.

I hereby declare:
The above information is correct. In particular, I am taking part in the study voluntarily and I know that I can withdraw my consent to participate at any time without giving reasons and cancel or terminate my participation without incurring any personal disadvantages.

I agree that the SMART-AGE research team may contact me during the study phase, for example if technical problems occur, and may also contact me after the end of my participation in the study until the end of the project (March 31, 2026) for further questions.

I expressly consent to the processing of my personal data in the context of the above-mentioned research project, as explained in the information sheet and the privacy policy, to the following extent.

1. I consent to my personal data (name, date of birth, address, telephone number, email address) and my other data (see list below) being recorded and stored pseudonymously (i.e. without mentioning my name or other identifying features) and processed for the anonymized evaluation of the results as part of the study. This also includes the processing of special categories of personal data pursuant to Art. 9 (1) GDPR in the form of health data.

2. I agree to the collection and anonymized evaluation of the following data categories:

a. Demographic data (e.g. marital status, education, family circumstances, health, income, housing situation, social participation, environment, leisure activities), collected through questionnaires
b. Data on physical function and performance (e.g. walking speed), recorded by questionnaires, motor tests and activity sensor
c. Psychological data (e.g. tests of mental abilities), collected through questionnaires/tests

3. I agree that my anonymized data may be used for non-commercial evaluations and for scientific publications.

4. I consent to my research data being made publicly accessible in anonymous form for scientific use via storage media (so-called repositories) in the event of publications, so that research results can be independently verified.
   Furthermore, the data provided by me or collected as part of the study will primarily be used for the questions presented in this information document. In the future, however, further analyses with these data may become necessary, which will be dealt with in the context of other research projects; these will then be used exclusively in anonymized form.
   The purpose, type and scope of subsequent use by third parties cannot be specifically foreseen at the present time. These future research projects will be discussed separately by the relevant ethics committee. I will not be required to provide further information or consent.

5. I agree that a voice recording will be made during a cognitive testing task as part of the study. This serves to facilitate the recording and evaluation of the task. The voice recording will only be stored for the day of the survey and will be deleted as soon as the employees have compared it with the recorded notes.

Please check the box: yes ☐ no ☐

6. I would like to be informed about changes or abnormalities.

Please check the box: yes ☐ no ☐

The granting of this consent to participate in the study and to the processing of personal data is voluntary. Unless consent is revoked, it is valid for an unlimited period of time. Consent can be withdrawn at any time with effect for the future. It can also be revoked in relation to individual data. Should I wish to change my decision at a later date, I will inform the study team immediately. If consent is refused or withdrawn, I will not suffer any disadvantages as a result.

The withdrawal of consent should be addressed to: **see contact and contact person.**

Data protection

I have been informed and voluntarily agree that my data collected in the study, in particular information about my health, may be recorded in pseudonymized form for the purposes described in the information leaflet, evaluated and, if necessary, passed on in anonymized form. Third parties will not have access to personal documents. The personal data will be anonymized as soon as this is possible in accordance with the research purpose. The results of the study will only be published in anonymized form. The data will be stored for 10 years after completion of the study. I am aware that this consent can be revoked at any time in writing or verbally without giving reasons and without any disadvantages for me. This does not affect the legality of the data processing carried out up to the point of withdrawal. When withdrawing consent, please note that my data can only be deleted until it has been anonymized, after which the data can no longer be associated with me personally.

Place, date: ___________________________________

____

Signature of study partner

Informing person

I have informed the study partner about the aim and procedure of the study as well as about the risks during an interview. I have given the study partner a copy of the information sheet and the consent and informed consent form.

____

Place, date | Surname, first name of the person providing information (in block capitals)

____

Signature of the informing person

Contact and contact person:

Project coordination:

Dr. Tobias Eckert<br>Dr. Claudia Hellmund	Heidelberg University - Network Aging Research (NAR)<br>Bergheimer Straße 20 - 69115 Heidelberg<br>Phone: 06221 - 54 8992<br>Availability: Mon 14:00-17:00 + Wed/Fri 09:00-12:00<br>Email: smartage@nar.uni-heidelberg.de

Project management:

Prof. Dr. Jürgen Bauer	Geriatric Center of the University Hospital Heidelberg<br>Agaplesion Bethanien Hospital Heidelberg<br>Rohrbacher Straße 149 - 69126 Heidelberg<br>Phone: 06221 - 319 1501
Prof. Dr. Hans-Werner Wahl	Heidelberg University - Network Aging Research (NAR)<br>Bergheimer Straße 20 - 69115 Heidelberg
Prof. Dr. Barbara Paech	Heidelberg University - Institute for Computer Science and Software Engineering<br>Im Neuenheimer Feld 205 - 69120 Heidelberg
Prof. Dr. Lorenzo Masia	Heidelberg University - Central Institute for Computer Engineering (ZITI)<br>Im Neuenheimer Feld 368 - 69120 Heidelberg

- For Your Records -

Detailed Data Protection Information

With the following information, we provide you with a detailed overview of the processing of your personal data within the scope of the above-mentioned research purpose and your rights under data protection law.

1. Personal Data

To achieve the scientific research purpose of the SMART-AGE study, it is necessary to process your personal data. Personal data, according to Article 4(1) of the General Data Protection Regulation (GDPR), is any information relating to an identified or identifiable natural person.

2. Controller and Data Protection Officer

The controller responsible for the processing of personal data is:

Project Lead SMART-AGE, Prof. Dr. Jürgen Bauer, Rohrbacher Straße 149, 69126 Heidelberg; Prof. Dr. Lorenzo Masia, Im Neuenheimer Feld 368, 69120 Heidelberg; Prof. Dr. Barbara Paech, Im Neuenheimer Feld 205, 69120 Heidelberg; Prof. Dr. Hans-Werner Wahl, Bergheimer Straße 20, 69115 Heidelberg; Email: smartage@nar.uni-heidelberg.de

The Data Protection Officer of the University of Heidelberg is:

Ass. jur. Christoph Wassermann
Seminarstr. 2
69117 Heidelberg
Tel. +49 6221 54-12070
Email: datenschutz@uni-heidelberg.de

3. Purpose of Data Processing

We process your personal data exclusively for a specified, clear, and legitimate purpose. The research purpose and goal of the SMART-AGE study is to determine what improvements in quality of life, social integration, and attention to personal health can be achieved through the use of a set of interconnected apps.

4. Legal Basis for Data Processing and Withdrawal of Consent

The legal basis for the processing of your personal data is your consent pursuant to Art. 6 (1) and (1) (a) GDPR, if and insofar as you give it.

You can revoke your consent at any time in its entirety or in relation to individual data with effect for the future. Effect for the future means that the withdrawal of consent does not affect the lawfulness of processing based on consent before its withdrawal.

5. Processing of personal data

Your personal data will be processed exclusively to achieve the above-mentioned scientific research purpose.

We will anonymize your personal data as soon as this is possible according to the research purpose. After completion of the study in April 2026, the link between the number assigned to you (pseudonym) and your personal data (e.g. telephone number, address) will be deleted. The data will then only be available in anonymized form.

Until it is possible to anonymize your data (this takes place at the end of the study), it will only be processed in pseudonymized form. Pseudonymization means that the data collected is marked with a number/letter code instead of the name/date of birth etc. The data can then only be identified by certain persons. The data can then only be assigned to a specific person by certain authorized persons with additional information (so-called "key").

6. Recipients

Within Heidelberg University and the other participating universities, your personal data will only be forwarded to the researchers involved in the above-mentioned study. Within the circle of the above-mentioned internal recipients, your personal data will only be forwarded if and insofar as this is necessary to achieve the scientific research purpose (e.g. for further processing of the data).

Your personal data will not be transferred to third parties. Data will only be transferred to third parties in cases where Heidelberg University is legally obliged to do so. Outside of Heidelberg University's legal obligations, we will only transfer data that does not allow any reference to your person, especially in the case of scientific publication of the results of the study. The data is anonymized, for example, by aggregating it and deleting the personal reference at the end of the study.

Personal data is not transferred to third countries outside the EU or the EEA or to an international organization.

7. Automated decision-making

Automated decision-making, including profiling, does not take place.

8. Storage Period

Personal data will be deleted in accordance with data protection regulations. In particular, they will be deleted when they are no longer necessary for the purposes for which they were collected or otherwise processed.

After completion of the study on March 31, 2026, the link between the number assigned to you (pseudonym) and your personal data (e.g. telephone number, address) will be deleted. The data will then only be available in anonymized form.

9. Rights

You have the following rights in relation to personal data concerning you:

- Right to confirmation as to whether data concerning you is being processed and to information about the processed data, to further information about the data processing and to copies of the data (Art. 15 GDPR),
- Right to rectification or completion of incorrect or incomplete data (Art. 16 GDPR),
- Right to immediate deletion of the data concerning you (Art. 17 GDPR),
- Right to restriction of processing (Art. 18 GDPR),
- Right to receive the data concerning you and provided by you and to transmit this data to other controllers (Art. 20 GDPR),

According to § 13 para. 4 LDSG are

- the right of access pursuant to Art. 15 GDPR,
- the right to rectification pursuant to Art. 16 GDPR,
- the right to restriction of processing pursuant to Art. 18 GDPR and
- the right to object pursuant to Art. 21 GDPR

to the extent that these rights are likely to render impossible or seriously impair the achievement of the research purpose and the restriction is necessary for the fulfillment of the research purpose. Furthermore, the right of access pursuant to Article 15 GDPR does not exist if the data is necessary for the purposes of scientific research and the provision of information would require a disproportionate effort.

Right to Objection

You also have the right to object, on grounds relating to your particular situation, at any time to processing of personal data concerning you which is based on point (e) or (f) of Article 6(1), including profiling based on those provisions. This right exists in accordance with Section 13 (4) LDSG.

You also have the right to complain to the supervisory authority about the processing of your personal data by Heidelberg University (Art. 77 GDPR). The supervisory authority within the meaning of Article 51 (1) GDPR for Heidelberg University is, in accordance with Section 25 (1) LDSG:

The State Commissioner for Data Protection and Freedom of Information Baden-Württemberg

House address: Lautenschlagerstraße 20
70173 Stuttgart

Postal address: P.O. Box 10 29 32
70025 Stuttgart

Phone: 0711/615541-0
Fax: 0711/615541-15
E-mail: poststelle@lfdi.bwl.de

10. Contact Addresses for Further Inquiries

Project coordination:

Dr. Tobias Eckert
Dr. Claudia Hellmund
Heidelberg University - Network Aging Research (NAR)
Bergheimer Straße 20 - 69115 Heidelberg
Phone: 06221 - 54 8992
Availability: Mon 14:00-17:00 + Wed/Fri 09:00-12:00
Email: smartage@nar.uni-heidelberg.de

Project management:

Prof. Dr. Jürgen Bauer
Geriatric Center of the University Hospital Heidelberg
Agaplesion Bethanien Hospital Heidelberg
Rohrbacher Straße 149 - 69126 Heidelberg
Phone: 06221 - 319 1501

Prof. Dr. Hans-Werner Wahl
Heidelberg University - Network Aging Research (NAR)
Bergheimer Straße 20 - 69115 Heidelberg

Prof. Dr. Barbara Paech
Heidelberg University - Institute for Computer Science and Software Engineering
Im Neuenheimer Feld 205 - 69120 Heidelberg

Prof. Dr. Lorenzo Masia
Heidelberg University - Central Institute for Computer Engineering (ZITI)
Im Neuenheimer Feld 368 - 69120 Heidelberg

Declaration of consent for participation in the SMART-AGE study after group allocation

I agree to participate in the study.

________________________, ________________________
(surname) (first name)

born on ______________________,

I am aware that I was randomly assigned to one of the three study groups after the first home visit. The study groups differ in terms of the number of home visits and the apps provided. It is not possible to switch to a study group other than the one randomly assigned.

I know that I will not incur any costs by participating in the study. I also know that the study will run for a maximum of one year.

The tablet required for the study will become my property if I have participated in the study for the entire study period.

I also agree to pseudonymized recording and anonymized analysis of my data collected in the study (see declaration of consent for study participation).

This also includes pseudonymized usage data of the apps provided as part of the study:

a) Usage data (e.g. how often the tablet is used, IP addresses), automatically recorded based on the use of the tablet and its applications (apps)
b) Health, exercise and activity data (e.g. information on diet, physical activity, performance of strength and balance exercises) by self-reporting in the apps used by SMART-AGE (smartIMPULS, KOKU)

I will receive a copy of the declaration of consent.

As part of the survey, changes and possible indications of health problems can be noted on the basis of the results. I am free to decide whether these are discussed with me or not ("right to remain ignorant").
If I wish to be informed about this, I will be told how to proceed. I am usually advised to discuss this with my family doctor. The study staff will not contact my family doctor for this purpose. This is exclusively my right.

Data Protection

I have been informed and voluntarily agree that the data collected in the study, particularly information about my health, can be recorded, evaluated, and possibly also shared in pseudonymized form for the purposes described in the information sheet. Third parties will not have access to personal documents. Personal data will be anonymized as soon as possible according to the research purpose. Any publication of the study results will be done exclusively in anonymized form. The data will be retained for 10 years after the study concludes. I am aware that this consent can be withdrawn at any time, either in writing or orally, without any disadvantages to me. The legality of data processing up to the point of withdrawal will not be affected. Please note that upon withdrawal of consent, your data can only be deleted up to the point of their anonymization; thereafter, the data cannot be associated with my person.

I hereby declare:
The above information is accurate, I voluntarily participate in the study, and I know that I can withdraw my consent to participate at any time without giving reasons and can discontinue or end my participation without any personal disadvantages.

1. I agree that my anonymized data may be used for non-commercial evaluations and scientific publications.

2. I agree that my research data may be made publicly accessible in anonymous form for scientific use (e.g., through repositories) in publications so that research results can be independently verified.

yes ☐ no ☐

I would like to be informed about changes or abnormalities.

yes ☐ no ☐

The withdrawal of consent should be addressed to: **see contact and contact person.**

Place, date   Surname, first name of the study partner

Signature of study partner

Contact and contact person:

Project coordination:

Dr. Tobias Eckert<br>Dr. Claudia Hellmund	Heidelberg University - Network Aging Research (NAR)<br>Bergheimer Straße 20 - 69115 Heidelberg<br>Phone: 06221 - 54 8992<br>Availability: Mon 14:00-17:00 + Wed/Fri 09:00-12:00<br>Email: smartage@nar.uni-heidelberg.de

Project management:

Prof. Dr. Jürgen Bauer	Geriatric Center of the University Hospital Heidelberg<br>Agaplesion Bethanien Hospital Heidelberg<br>Rohrbacher Straße 149 - 69126 Heidelberg<br>Phone: 06221 - 319 1501
Prof. Dr. Hans-Werner Wahl	Heidelberg University - Network Aging Research (NAR)<br>Bergheimer Straße 20 - 69115 Heidelberg
Prof. Dr. Barbara Paech	Heidelberg University - Institute for Computer Science and Software Engineering<br>Im Neuenheimer Feld 205 - 69120 Heidelberg
Prof. Dr. Lorenzo Masia	Heidelberg University - Central Institute for Computer Engineering (ZITI)<br>Im Neuenheimer Feld 368 - 69120 Heidelberg

Declaration of consent for participation in the SMART-AGE study after group allocation

I agree to participate in the study.

_________________________, _________________________
(surname) (first name)

born on ______________________,

I am aware that I was randomly assigned to one of the three study groups after the first home visit. The study groups differ in terms of the number of home visits and the apps provided. It is not possible to switch to a study group other than the one randomly assigned.

I know that I will not incur any costs by participating in the study. I also know that the study will run for a maximum of one year.

The tablet required for the study will become my property if I have participated in the study for the entire study period.

I also agree to pseudonymized recording and anonymized analysis of my data collected in the study (see declaration of consent for study participation).

This also includes pseudonymized usage data of the apps provided as part of the study:

a) Usage data (e.g. how often the tablet is used, IP addresses), automatically recorded based on the use of the tablet and its applications (apps)

I will receive a copy of the declaration of consent.

As part of the survey, changes and possible indications of health problems can be noted on the basis of the results. I am free to decide whether these are discussed with me or not ("right to remain ignorant").
If I wish to be informed about this, I will be told how to proceed. I am usually advised to discuss this with my family doctor. The study staff will not contact my family doctor for this purpose. This is exclusively my right.

Data Protection

I have been informed and voluntarily agree that the data collected in the study, particularly information about my health, can be recorded, evaluated, and possibly also shared in pseudonymized form for the purposes described in the information sheet. Third parties will not have access to personal documents. Personal data will be anonymized as soon as possible according to the research purpose. Any publication of the study results will be done exclusively in anonymized form. The data will be retained for 10 years after the study concludes. I am aware that this consent can be withdrawn at any time, either in writing or orally, without any disadvantages to me. The legality of data processing up to the point of withdrawal will not be affected. Please note that upon withdrawal of consent, your data can only be deleted up to the point of their anonymization; thereafter, the data cannot be associated with my person.

I hereby declare:
The above information is accurate, I voluntarily participate in the study, and I know that I can withdraw my consent to participate at any time without giving reasons and can discontinue or end my participation without any personal disadvantages.

1. I agree that my anonymized data may be used for non-commercial evaluations and scientific publications.

2. I agree that my research data may be made publicly accessible in anonymous form for scientific use (e.g., through repositories) in publications so that research results can be independently verified.

yes ☐ no ☐

I would like to be informed about changes or abnormalities.

yes ☐ no ☐

The withdrawal of consent should be addressed to: **see contact and contact person.**

Place, date Surname, first name of the study partner

Signature of study partner

Contact and contact person:

Project coordination:

Dr. Tobias Eckert<br>Dr. Claudia Hellmund	Heidelberg University - Network Aging Research (NAR)<br>Bergheimer Straße 20 - 69115 Heidelberg<br>Phone: 06221 - 54 8992<br>Availability: Mon 14:00-17:00 + Wed/Fri 09:00-12:00<br>Email: smartage@nar.uni-heidelberg.de

Project management:

Prof. Dr. Jürgen Bauer	Geriatric Center of the University Hospital Heidelberg<br>Agaplesion Bethanien Hospital Heidelberg<br>Rohrbacher Straße 149 - 69126 Heidelberg<br>Phone: 06221 - 319 1501
Prof. Dr. Hans-Werner Wahl	Heidelberg University - Network Aging Research (NAR)<br>Bergheimer Straße 20 - 69115 Heidelberg
Prof. Dr. Barbara Paech	Heidelberg University - Institute for Computer Science and Software Engineering<br>Im Neuenheimer Feld 205 - 69120 Heidelberg
Prof. Dr. Lorenzo Masia	Heidelberg University - Central Institute for Computer Engineering (ZITI)<br>Im Neuenheimer Feld 368 - 69120 Heidelberg
